# Single‐Position Peptide Clustering for Peptidomics Reveals Novel Disease Biomarkers and Dysregulated Proteolytic Characteristics

**DOI:** 10.1002/advs.202510910

**Published:** 2025-11-18

**Authors:** Na Li, Yaxin Zhu, Yumeng Yan, Jifeng Wang, Lili Niu, Xiang Ding, Mengmeng Zhang, Zhensheng Xie, Tanxi Cai, Xiaojing Guo, Jianming Luo, Peng An, Xiangqian Guo, Fuquan Yang

**Affiliations:** ^1^ Laboratory of Proteomics, Institute of Biophysics Chinese Academy of Sciences Beijing 100101 China; ^2^ University of Chinese Academy of Sciences Beijing 100049 China; ^3^ Department of Pediatrics The First Affiliated Hospital of Guangxi Medical University Nanning 530021 China; ^4^ Department of Nutrition and Health China Agricultural University Beijing 100193 China; ^5^ Henan Provincial Engineering Center for Tumor Molecular Medicine Zhongyuan Intelligent Medical Laboratory School of Basic Medical Sciences Henan University Kaifeng 475004 China

**Keywords:** amino acid score, biomarker, peptidomics, peptide cluster, single‐position peptide clustering

## Abstract

Mass spectrometry‐based peptidomics provides a comprehensive platform for mapping global proteolytic alterations and identifying disease biomarkers. However, existing analytical frameworks often lack the precision to capture disease‐specific signatures. Here, a single‐position peptide clustering strategy is introduced, leveraging the amino acid score (aa‐score) method, and applying it to plasma peptidomics in β‐thalassemia. By integrating grouped aa‐scores with tailored visualization, a clear and interpretable profile of protein degradation is generated from otherwise redundant datasets. Importantly, the use of heavy‐labeled peptides or reference samples in targeted quantitative peptidomics enabled, for the first time, the proposal of aa position‐based peptide cluster biomarkers. Combined with proteomics and complementary analyses, this strategy revealed disease‐specific peptide‐protein‐protease relationships. Furthermore, the robustness of the aa‐score framework is demonstrated by applying an individualized algorithm based on reference samples in an independent cohort study, highlighting its capacity to address missing values and improve overall performance.

## Graphical abstract

A novel amino acid (aa)‐score‐based single‐position peptide clustering strategy is developed for peptidomics, enabling precise profiling of protein proteolysis in plasma from β‐thalassemia cohort. The method identifies new aa position‐based peptide cluster biomarkers validated by heavy‐labeled peptides, visualizes aggregated changes, uncovers disease‐specific peptide‐protein‐protease features, and improves cohort study performance through reference sample‐assisted individualized algorithms.

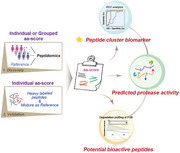

## 1 Introduction

Once regarded merely as a subset of the proteome, the peptidome is now recognized as a distinct molecular entity with unique biological relevance.^[^ ^1^ ^]^ Endogenous peptides arise either through proteolytic processing of precursor proteins or via direct translation from small open reading frames.^[^ ^2^, ^3^ ^]^ While some peptides, such as neuropeptides,^[^ ^4^ ^]^ exert defined physiological functions, most have historically been dismissed as metabolic byproducts or “waste” due to their apparent lack of bioactivity. Yet, these peptides represent a vast reservoir of potential biomarkers with diagnostic and therapeutic value.^[^ ^5^ ^]^

The amino acid (aa) composition of peptide sequences encodes information on the proteolytic mechanisms driving their generation. Even in the absence of known biological functions, these peptides act as readouts of upstream enzymatic activities. Despite its centrality in physiology and pathology, proteolysis remains poorly understood, largely because many proteases, their substrate specificities, and their regulatory networks remain uncharacterized.^[^ ^6^, ^7^ ^]^ However, protease activity can often be inferred from cleavage products based on the fundamental principles of protease action. Such indirect profiling provides critical insights into disease mechanisms and highlights potential therapeutic targets.^[^ ^8^ ^]^

Peptidomics occupies a unique niche among omics sciences. Specific proteolytic cleavage events and exopeptidase activities generate gradient‐like, apparently redundant peptide data. Endogenous peptides’ intrinsic features—low abundance, chemical heterogeneity, and detection challenges—compound variability in identification, inter‐individual differences, and extensive missing values.^[^ ^9^ ^]^ Current analytical methods, largely adapted from proteomics,^[^ ^10^ ^]^ are inadequate for capturing the specific information encoded in peptidomic data. Thus, novel data analysis strategies tailored to peptidomics are essential for unlocking their full clinical potential.

β‐thalassemia, a hemoglobinopathy caused by mutations in the hemoglobin subunit beta (*HBB*) gene, has been associated with significant alterations in red blood cells and plasma composition. Enhanced proteolysis in thalassemic erythroid cells has long been observed,^[^ ^11^ ^]^ and protease activity has been linked to disease severity.^[^ ^12^ ^]^ Protein quality‐control pathways, particularly the proteasome and autophagy, are implicated as potential therapeutic targets.^[^ ^13^ ^]^ Our plasma proteomics studies revealed that among the top five discriminant proteins, aside from ferritin subunits, the remaining candidates—platelet‐activating factor acetylhydrolase and cathepsin S—are proteases.^[^ ^14^ ^]^ Additional proteases, particularly lysosomal enzymes, are upregulated in β‐thalassemia. In extracellular vesicle proteomics, complement C1s, another protease, was identified in our diagnostic protein panel.^[^ ^15^ ^]^ Moreover, inhibition of matriptase‐2 (TMPRSS6), a type II transmembrane serine protease, increases hepcidin (HAMP) expression and alleviates multiple β‐thalassemia symptoms.^[^ ^16^ ^]^ Collectively, these findings highlight protease networks as central yet underexplored mediators of β‐thalassemia pathology.

Here, we introduce a single‐position peptide clustering strategy to systematically analyze redundant peptides generated by enzymatic activity at specific aa sites within proteins. Using mass spectrometry (MS)‐based peptidomics and the aa score (aa‐score) framework, we constructed a data ecosystem encompassing concepts such as protein aa positions, peptide cluster biomarkers, and visualization techniques. Applied to plasma samples from β‐thalassemia cases (minor [TT], intermediate [TI], and major [TM]) and healthy controls (Ctr), this strategy uncovered candidate screening biomarkers and distinct disease characteristics. Finally, we refined the approach and validated its superiority in handling missing values and enhancing data utilization in an independent cohort of colorectal cancer (CRC) patients.

## 2 Results

### 2.1 Overview of the Plasma Peptidomic Study of β‐Thalassemia

**Figure 1:**
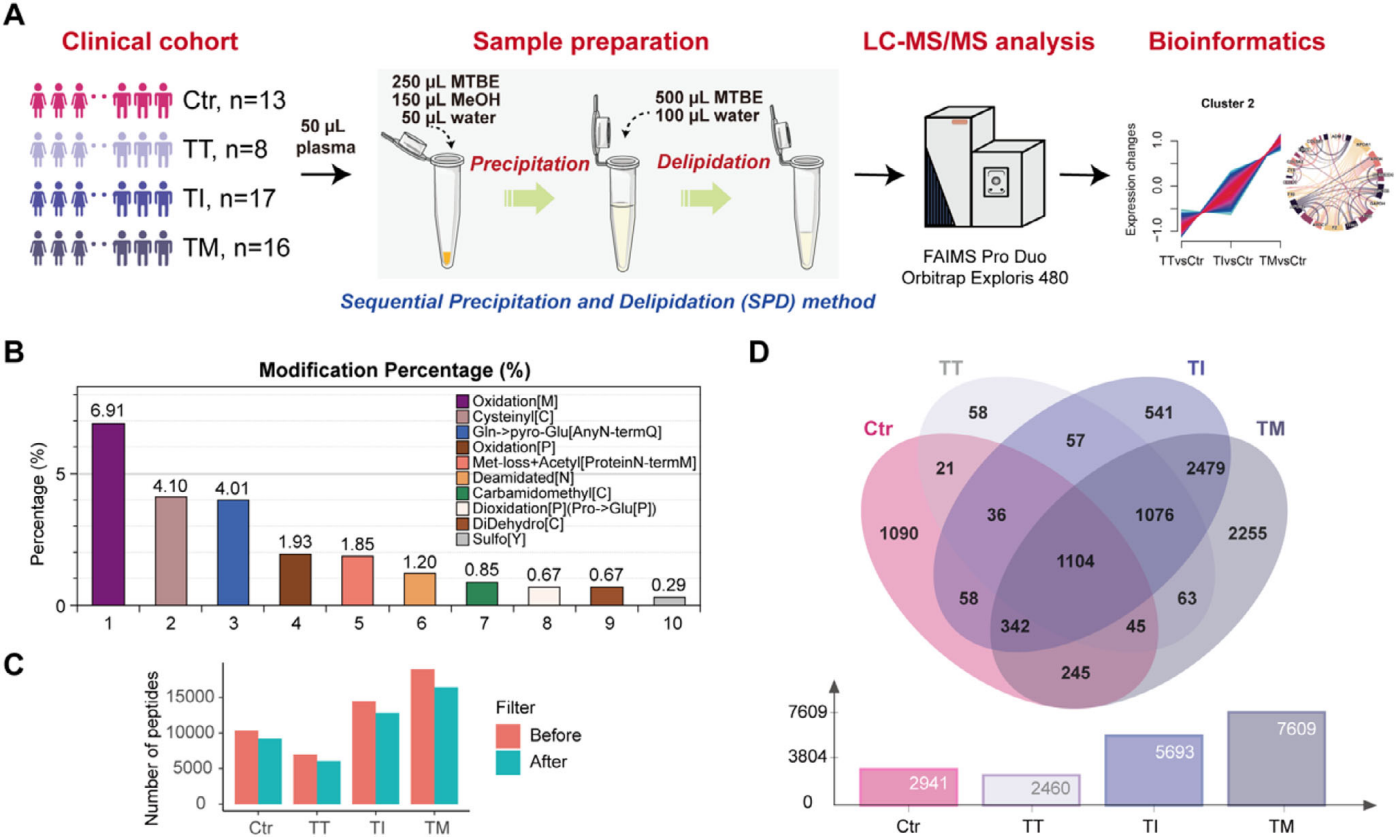
Unbiased peptidomic profiling of a β‐thalassemia cohort. A) Clinical cohort composition and workflow. B) Peptide modification distribution highlighting methionine oxidation, cysteinylation, and N‐terminal pyro‐glutamate formation. C) Comparison of peptide numbers before and after MS/MS quality filtering. D) Venn diagram of unique and shared peptides across Ctr, TT, TI, and TM, with bar graph of total peptide counts.

Plasma samples from 8 TT, 17 TI, 16 TM, and 13 Ctr individuals were analyzed by quantitative plasma peptidomics. Plasma peptides were enriched using the sequential precipitation and delipidation (SPD) method,^[^ ^17^ ^]^ optimized for large‐scale clinical peptidomic studies. The overall workflow is shown in **Figure** **1**A.

Before conducting database searches, we performed an open search to assess the types of peptide modifications. Common modifications—including methionine oxidation (M), cysteinylation of cysteine (C), and N‐terminal glutamine conversion to pyro‐glutamate (Gln→pyro‐Glu[AnyN‐termQ])—were then set as dynamic modifications in subsequent searches using Proteome Discoverer 2.4 (Figure 1B). Although some peptides were confidently identified at false discovery rate (FDR) < 0.01, their MS/MS spectra lacked sufficient quality (Figure S1A, Supporting Information). To improve reliability, only peptides with at least four consecutive b‐ or y‐ions were retained. This quality filter removed ≈10.7–13.4% of peptides across groups (Figure 1C). Additionally, many peptides were detected in only one or two samples per group (Figure S1B, Supporting Information). To minimize bias from low‐abundance events, only peptides present in at least three samples per group were included in the downstream analysis.

After stringent screening, 9470 peptides were identified across the cohort: 2941 in Ctr, 2460 in TT, 5693 in TI, and 7609 in TM (Figure 1D; Data S1, Supporting Information). Only 1104 peptides (11.7%) were shared across all four groups. To trace tissue‐ or cell‐specific origins, the corresponding proteins were annotated with PaGenBase. As disease severity increased, peptides of blood origin became more prominent (Figure S1C, Supporting Information), consistent with the severity of hemolysis—a hallmark of β‐thalassemia.

### 2.2 Traditional Differential Peptide Analysis and the Introduction of the Amino Acid Score

**Figure 2:**
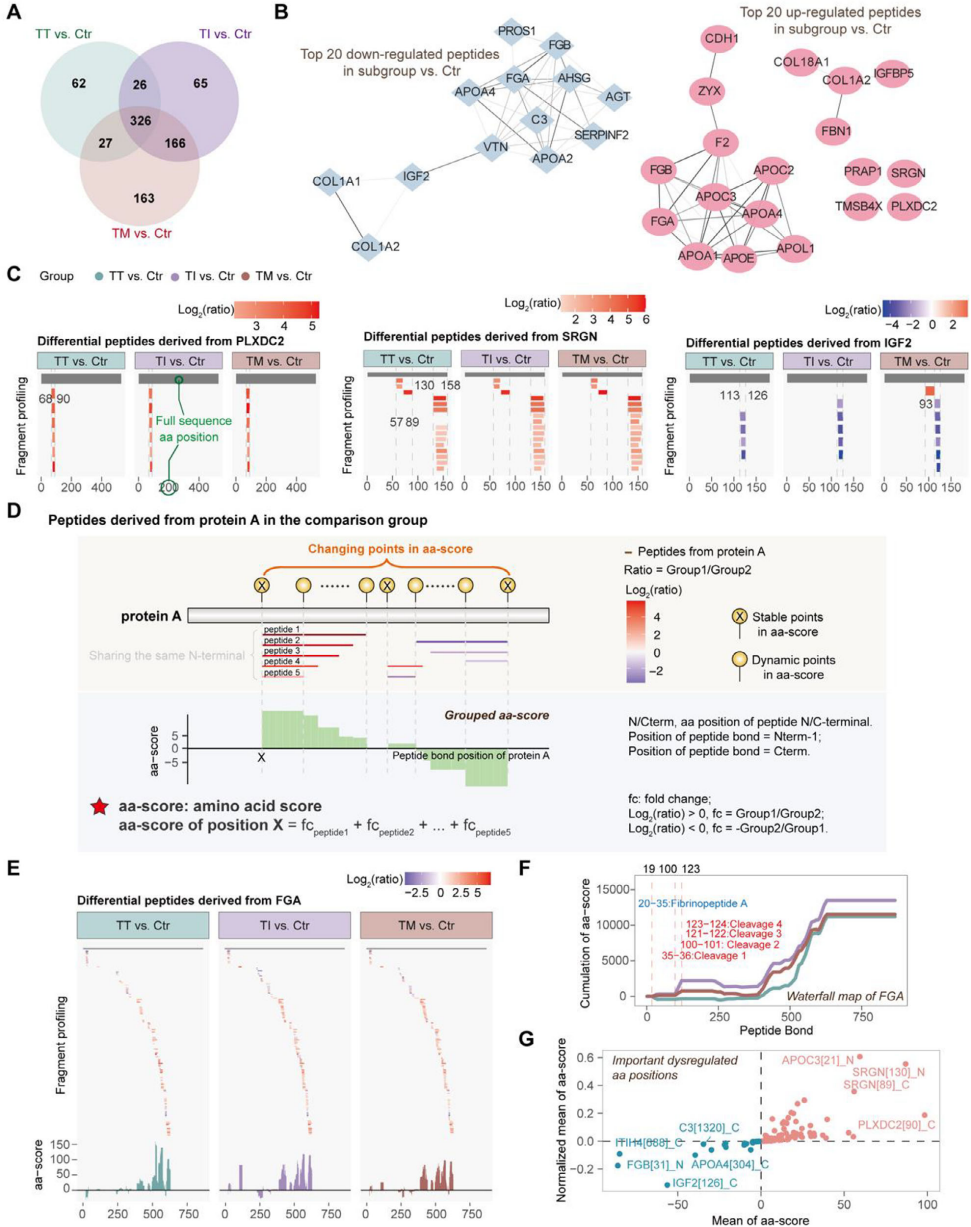
Introduction of the aa‐score algorithm and its application to β‐thalassemia. A) Overlap of differential peptides across subgroups relative to Ctr. B) Protein‐protein interaction network of proteins corresponding to the top 20 differential peptides, ranked by fold change in each subgroup relative to Ctr. C) Fragment profiling of differential peptides from PLXDC2, SRGN, and IGF2, with color mapped to the log_2_‐transformed fold changes. D) Schematic of the grouped aa‐score algorithm, which quantifies cumulative fold changes between high‐ and low‐intensity peptides across two groups, categorizing clustered differential peptides as “stable” or “dynamic” points. E) Fragment profiling of differential peptides derived from FGA and aa‐score of specific positions. F) Waterfall map of FGA, highlighting UniProt‐annotated cleavage sites and peptide. Colored segments represent UniProt annotations. Black peptide bond position numbers indicate turning points in cumulative grouped aa‐score curves corresponding to annotated UniProt aa sites. G) Critical dysregulated aa positions in proteins linked to β‐thalassemia, showing enhanced (red) or reduced (blue) proteolytic activity. Mean aa‐score was normalized by the number of peptide bonds per protein.

In label‐free quantitative peptidomics, 441, 583, and 682 differential peptides were identified in TT vs Ctr, TI vs Ctr, and TM vs Ctr, respectively (**Figure** **2**A; Data S2, Supporting Information). Top 20 up‐ or down‐regulated unique peptides largely originated from fibrinogen, complement proteins, collagen, lipoproteins, and insulin‐like growth factors. Their protein–protein interactions are shown in Figure 2B.

Among up‐regulated peptides, all unique fragments from plexin domain‐containing protein 2 (PLXDC2) ranked at the top, clustering within aa [68‐90]. Similarly, the significantly up‐regulated peptides from serglycin (SRGN) consistently marked cleavage hotspots at [57‐89] and [130‐158]. In contrast, down‐regulated insulin‐like growth factor II (IGF2) peptides shared a conserved C‐terminus (Figure 2C). These results indicate that peptide clusters arising from specific protein loci may carry stronger diagnostic potential than individual peptides. However, proteins producing numerous fragments, such as fibrinogen alpha chain (FGA), complicate analysis due to contradictory degradation trends and overlapping group‐specific degradation fragments. This complicates visualization and interpretation, making it challenging to distinguish biologically relevant proteolytic cleavage from nonspecific degradation.

To address this complexity, we developed the aa‐score method, which quantifies redundant peptides cumulatively mapped to the same protein site. This enables systematic aggregation of proteolytic diversity into single‐position peptide clusters. A major challenge is normalization across peptides with different MS response intensities. In untargeted peptidomics, where a reference sample is lacking, we introduced the grouped aa‐score, which represents the cumulative fold changes in peptide abundance between groups (Figure 2D). Fold changes may be positive or negative, indicating enhanced or reduced proteolysis at the given site. Only differential peptides contribute to this calculation. Shared aa sites across peptides are termed “stable points,” whereas non‐shared sites are “dynamic points”. Their orientation (N‐ or C‐terminus) reflects detected proteolytic product directionality. Collectively, both stable and dynamic points comprise “changing points” that reflect proteolytic variability.

For example, differential peptide profiling of FGA revealed extensive dysregulation (Figure 2E). Mapping fold changes using the aa‐score clarified these proteolytic hotspots. While plasma proteomics detected no overall change in FGA abundance between TM vs Ctr or TI vs Ctr, localized upregulation of segments [101‐122], [388‐442], [466‐483], and [495‐629] were evident, reflecting increased proteolysis. Proteins strongly associated with β‐thalassemia—hemoglobins, iron‐regulatory, and 13 candidate biomarkers from our earlier study^[^ ^2^ ^]^—were also examined. Surprisingly, no differential peptides were detected in most of these proteins, except HBB, hemoglobin subunit alpha (HBA1), and serotransferrin. Even here, only a few differential peptides were found, with reduced peptide numbers from HBB and HBA1 across β‐thalassemia subtypes (Figure S2A,B, Supporting Information).

To visualize proteolytic hotspots, particularly in the case of multiple pairwise comparisons, cumulative aa‐score profiles were mapped onto source proteins, producing waterfall maps. Notably, certain changing points in FGA, which served as turning points in the curves, aligned with the cleavage sites or the terminal of a peptide annotated in UniProt (Figure 2F). Thus, cumulative aa‐score profiling provides a powerful means of identifying functionally significant cleavage sites. Transition points—defined as changing points with directional shifts in score trends—were classified as disease‐associated important aa positions when consistently observed across all comparisons. As shown in Figure 2G, the most downregulated sites in β‐thalassemia included IGF2[126]_C, FGB[31]_N, ITIH4[688]_C, and APOA4[304]_C, while the most upregulated sites included SRGN[130]_N, APOC3[21]_N, SRGN[89]_C, and PLXDC2[90]_C.

### 2.3 Potential Peptide Cluster Biomarkers Based on AA‐Score for β‐Thalassemia Screening

**Figure 3:**
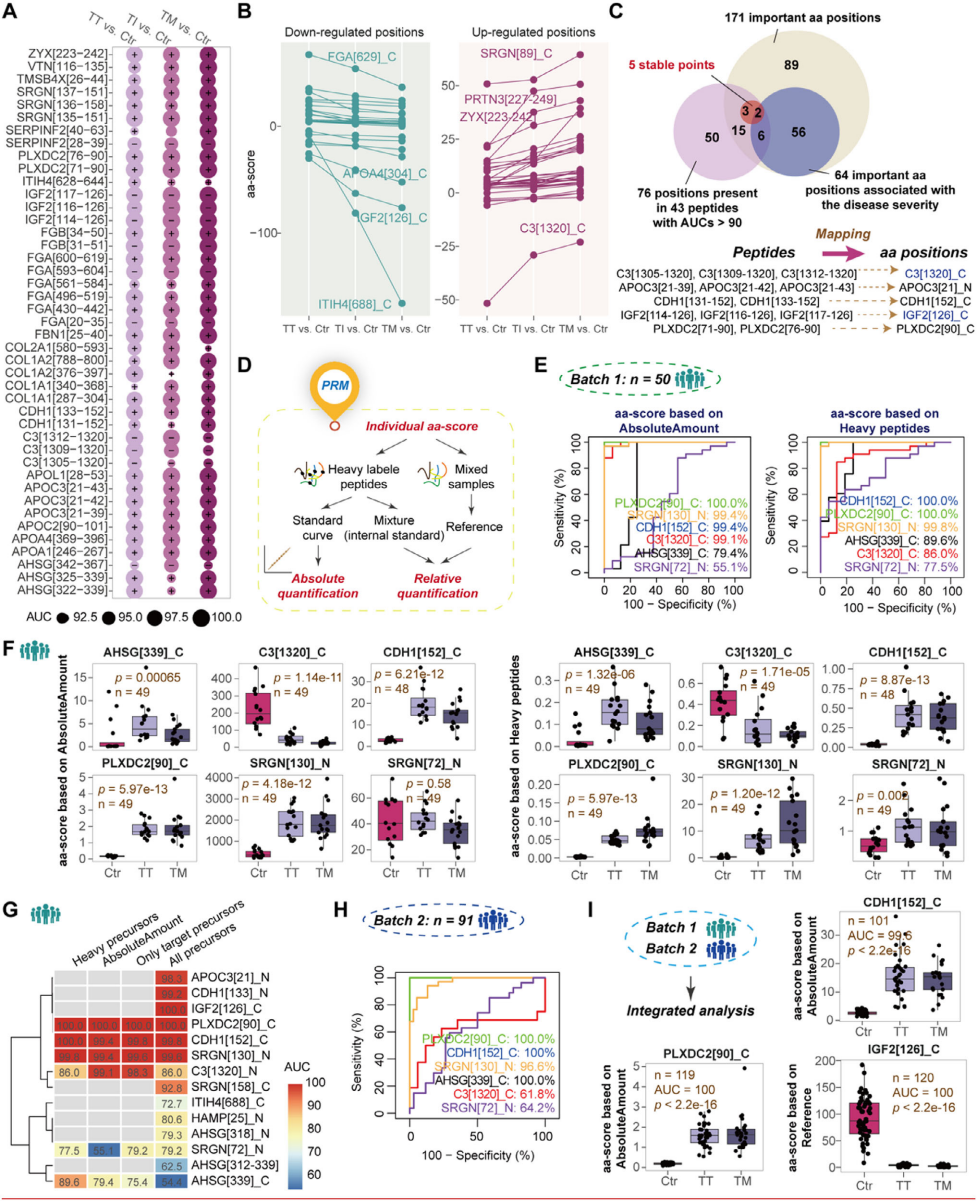
Identification and validation of aa positions with diagnostic potential for β‐thalassemia. A) ROC curves for differential peptides with AUC > 90% in all pairwise comparisons. “+” and “−” indicate up‐ and down‐regulated peptides, respectively. B) Trends in aa position changes across β‐thalassemia subtypes relative to Ctr. Blue and red represent the proteolytic activity that gradually decreases or increases, respectively, with the severity of the disease. The position suffix “_N” or “_C” designates the terminal exposure (N‐ or C‐terminus) of the proteolytic product. The suffix “_B” indicates sites where proteolytic products from both termini are simultaneously detected. C) Venn diagram showing overlap among aa positions in diagnostic peptides, important aa positions, and positions associated with disease severity. The relationship between peptides and their respective aa sites is illustrated. D) Workflow for calculating individual aa‐scores. E) ROC curves evaluating diagnostic potential of aa positions in Batch 1 PRM, with scores derived from absolute abundance (left) or heavy‐labeled internal standards (right). F) Boxplots of aa‐score distributions by absolute or relative quantification. G) Comparison of different calculation methods, including absolute quantification (AbsoluteAmount) and relative quantification with heavy‐labeled peptides (Heavy precursors) or reference samples (Only target precursors and All precursors), highlighting the robust performance of positions like PLXDC2[90]_C. H) ROC analysis of Batch 2 PRM. I) Integrated quantitative analysis across both PRM batches. “*n*” indicates the number of samples. Comparisons were performed between β‐thalassemia and Ctr, and reported *p*‐values were calculated using the Wilcoxon rank‐sum test. Boxplots show median (centerline), interquartile range (box), and 1.5× interquartile range (IQR) whiskers.

To identify candidate peptide biomarkers for β‐thalassemia, we performed Receiver Operating Characteristic (ROC) analyses on all differential peptides in each subgroup relative to Ctr. Peptides with an area under the curve (AUC) exceeding 90% in all comparisons were designated as potential screening biomarkers (**Figure** **3**A; Data S3, Supporting Information). A total of 43 peptides met this criterion, including APOL1[28‐53], FGB[31‐51], PLXDC2[71‐90], and PLXDC2[76‐90], which achieved perfect AUCs of 100%.

Across comparisons of TT vs Ctr, TI vs Ctr, and TM vs Ctr, we identified 27 progressively down‐regulated and 37 progressively up‐regulated important aa positions (Figure 3B). There were 26 aa positions overlapping between the 171 important aa positions and 76 aa positions mapped by the 43 peptides with diagnostic potential, of which 8 aa positions were associated with disease severity. Of the 26 shared positions, 5 were stable points within potential screening biomarkers (Figure 3C). Stable aa positions may provide a more reliable focus than single peptide, given the variability of non‐specific cleavage. These positions are initially derived from group comparisons (grouped aa‐score), but individual positions can be validated using heavy‐labeled peptides or reference standards.

For diagnostic validation, we employed parallel reaction monitoring (PRM) for targeted quantification of the analytes. To reduce the number of targeted peptides, only peptide with diagnostic potential were selected to represent specific aa positions, such as IGF2[114‐126], IGF2[117‐126], and IGF2[116‐126], which converged on the stable position IGF2[126]_C.

In the first PRM validation stage (Data S4, Supporting Information), individual aa‐scores were calculated using heavy‐labeled peptides or reference mixtures (Figure 3D). Two calculation methods were used with heavy‐labeled peptides: summing absolute quantification values or directly summing ratios of endogenous to labeled precursors (Figure 3E,F). Reference‐based calculations were performed using either only endogenous peptides corresponding to labeled peptides or all targeted representative peptides (Figure S3A–C, Supporting Information). Across methods, PLXDC2[90]_C and CDH1[152]_C showed the strongest diagnostic performance in Batch 1 PRM (Figure 3G), while IGF2[126]_C also distinguished β‐thalassemia from Ctr despite lacking heavy‐labeled synthesis. Batch 2 PRM analyses in an independent cohort (*n* = 91; Ctr: 40, TM: 12, TT: 39; Data S5, Supporting Information) confirmed that PLXDC2[90]_C, CDH1[152]_C, and IGF2[126]_C retained superior discriminatory power (Figure 3H; Figure S4A–D, Supporting Information). Integrated analysis across both batches validated the feasibility and robustness of the aa‐score‐driven diagnostic algorithm for clinical stratification (Figure 3I; Figure S4E, Supporting Information).

### 2.4 Specific Findings Using the AA‐Score Method

**Figure 4:**
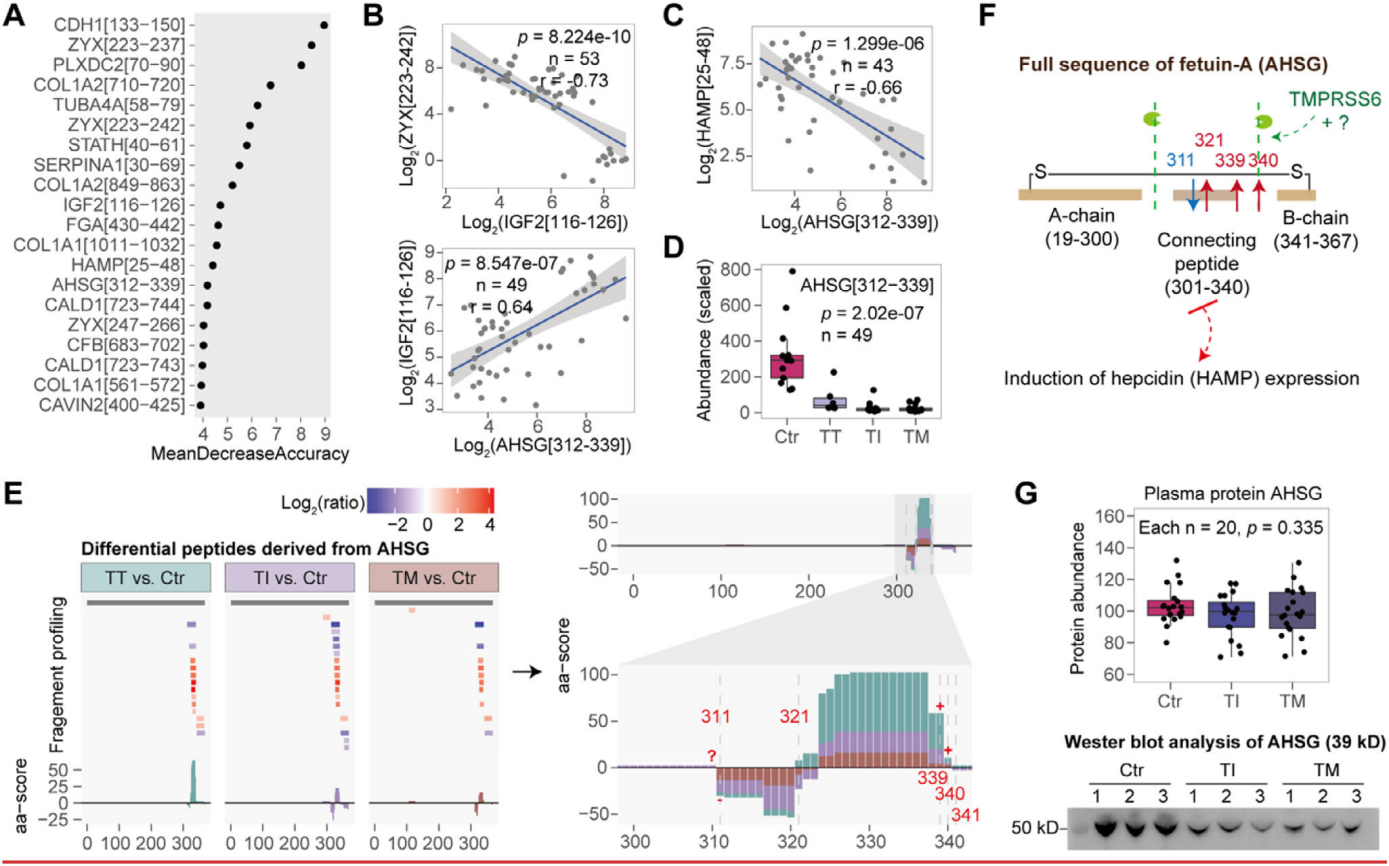
Discovery of dysregulated AHSG proteolysis. A) Random forest ranking of differential peptides influencing β‐thalassemia subgrouping, top 20 by mean decrease in accuracy. B) Correlation analysis among IGF2[116‐126], ZYX[223‐242], and AHSG[312‐339]. C) Strong negative correlation between AHSG[312‐339] and HAMP[25‐48]. D) Scaled abundance of AHSG[312‐339] across groups, highlighting significant downregulation. Comparisons between the β‐thalassemia and Ctr were analyzed using the Wilcoxon rank‐sum test. E) Fragment profiling and aa‐score analysis of AHSG, showing increased B‐chain production at bond positions 339 and 340. F) Schematic of AHSG proteolysis and its effect on hepcidin induction. Red arrows indicate enhanced cleavage; blue arrows indicate attenuated cleavage. G) Protein abundance and western blot analysis of AHSG. Boxplots show median (centerline), interquartile range (box), and 1.5× IQR whiskers. Statistical significance was assessed via one‐way ANOVA for the comparison of three groups. “*n*” indicates the number of samples.

Differential peptides overlapping across the three pairwise comparisons were subjected to random forest analysis to identify those most influential in β‐thalassemia subgrouping. Peptide importance was assessed by mean decrease accuracy, and the top 20 peptides were selected for subsequent correlation analysis (**Figure** **4**A; Figure S5A, Supporting Information). Notably, IGF2[116‐126], a segment within the preptin^[^ ^18^ ^]^ region, displayed correlations with multiple peptides, including those originating from zyxin (ZYX) and AHSG[312‐339] (Figure 4B; Figure S5B, Supporting Information). A strong negative correlation was observed between AHSG[312‐339] and HAMP[25‐48] in plasma samples (Figure 4C).

Fetuin‐A (AHSG) is a known substrate of TMPRSS6 in vitro, along with hemojuvelin and itself. The AHSG fragment [301‐340] acts as the linker between its A‐ and B‐chains. TMPRSS6‐mediated cleavage into two‐chain variants disrupts Fetuin‐A‐induced hepcidin expression.^[^ ^19^ ^]^ Among AHSG differential peptides, AHSG[312‐339] exhibited the most pronounced differences in TI vs Ctr and TM vs Ctr (Figure 4D), suggesting a potential inhibition of two‐chain processing and, consequently, enhanced Fetuin‐A‐mediated hepcidin induction. However, increased B‐chain production at bond positions 339 and 340, as indicated by the aa‐score, suggests that hepcidin induction may be attenuated in β‐thalassemia subgroups (Figure 4E,F). This reduction appears inversely proportional to disease severity, consistent with HAMP concentrations reported in stratified patient cohorts.^[^ ^20^, ^21^ ^]^ Proteomic analysis revealed no significant differences in AHSG abundance between patients and Ctr, yet western blotting confirmed downregulation of intact AHSG in β‐thalassemia, indicating disease‐specific proteolytic processing (Figure 4G).

### 2.5 Dysregulated Molecular Networks Composed of Protein, Protease, and Peptide

**Figure 5:**
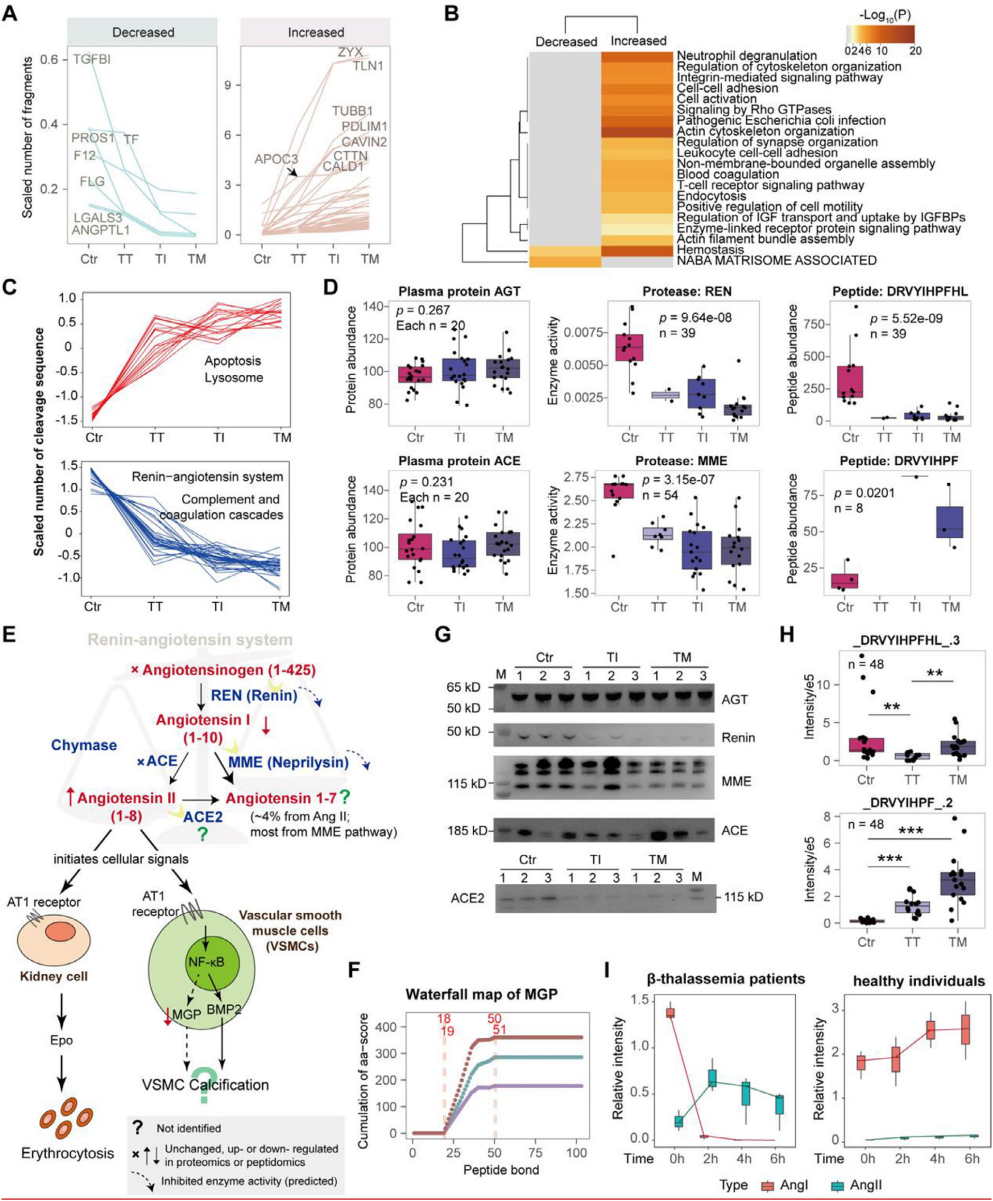
Protein degradation and protease prediction analyses. A) Proteins exhibiting progressive increases or decreases in the scaled number of identified peptides with disease severity. B) Pathway enrichment for proteins with altered peptide counts. C) Predicted protease pathway enrichment, with activity inferred from the scaled number of cleavage sequences identified by Proteasix. D) Scaled abundance of RAS‐associated proteins and proteases, alongside corresponding peptide changes. Comparisons across three or four groups were analyzed using one‐way ANOVA. E) Schematic linking RAS to erythrocytosis and vascular smooth muscle cell (VSMC) calcification, integrating cohort observations with literature evidence. Molecular changes observed in the β‐thalassemia cohort are annotated. Renin, REN; neprilysin, MME. F) Waterfall map of MGP. G) Western blot validation of RAS‐related protein abundance in plasma from β‐thalassemia patients and Ctr. Angiotensinogen, AGT. H) PRM quantification of AngI and AngII levels; boxplots illustrate relative intensities of precursors DRYIHPFHL_3 and DRYIHPF_2. Statistical significance was determined using *t*‐tests (*p < 0.05, **p < 0.01, and ***p < 0.001). I) Temporal dynamics of AngI substrate and AngII product following incubation with patient or Ctr plasma (each *n* = 3). Boxplots depict median (centerline), interquartile range (box), and 1.5× IQR whiskers; “*n*” indicates the number of samples.

Proteins with increased or decreased numbers of peptides were analyzed following normalization to sample number (**Figure** **5**A), Most of these proteins were degraded incrementally across five groups (from Ctr to TT, TI, TM), enriched in hemostasis and actin cytoskeleton organization pathways (Figure 5B). Proteases alterations were predicted via Proteasix, and a heatmap of scaled cleavage sequence counts per sample revealed distinct up‐ and down‐regulation patterns (Figure S6A, Supporting Information). Enzyme activity was inferred from scaled cleavage counts, allowing for classification into activated or inhibited enzymes, which correlated with disease severity. Activated enzymes were enriched in apoptosis, lysosome, and protein digestion and absorption pathways, whereas inhibited enzymes were linked to the renin‐angiotensin system (RAS), PID uPA‐uPAR pathway, NGF processing, and amelogenesis (Figure 5C; Figure S6B,C, Supporting Information).

Proteomic analysis revealed the coordinated upregulation of chaperone‐mediated autophagy (CMA) regulators, including heat shock cognate 71 kDa protein, lysosome‐associated membrane glycoprotein 2, and distinct heat shock protein 90 (HSP90) isoforms, alongside elevated lysosomal hydrolases and cofactors (Figure S6D, Supporting Information). Enhanced CMA may induce ferroptosis by disrupting lysosomal iron homeostasis and depleting antioxidants.^[^ ^22^ ^]^ CMA‐mediated ferritin degradation releases redox‐active iron (Fe^2^⁺/Fe^3^⁺), promoting reactive oxygen species generation via the Fenton reaction, exacerbating lipid peroxidation, and inducing lysosomal membrane permeabilization (LMP).^[^ ^23^ ^]^ Proteomic data indicated that LMP triggered cathepsin leakage and increased cytochrome c levels, signaling mitochondrial apoptosis (Figure S6E, Supporting Information). Although caspases were undetected in proteomics, peptidomic profiling confirmed their activation, suggesting enhanced apoptotic signaling (Figure S6F,G, Supporting Information).

Integrating proteomics, peptidomics, and literature, we mapped dysregulated RAS signaling in β‐thalassemia (Figure 5D,E). Angiotensin II (AngII), a key erythropoiesis regulator,^[^ ^24^, ^25^ ^]^ may contribute to elevated erythropoiesis. AngII also regulates matrix Gla protein (MGP) expression,^[^ ^26^ ^]^ reduced MGP carboxylation correlates with ectopic calcification in patients.^[^ ^27^ ^]^ Although MGP was undetected in proteomics, aa‐score analysis revealed increased degradation in β‐thalassemia (Figure 5F; Figure S6H, Supporting Information). Selected protein or peptide abundances were validated via western blot or PRM (Figure 5G,H). Angiotensin‐converting enzyme (ACE) protein levels remained unchanged in proteomics and western blotting, yet peptidomics revealed altered ACE activity, as inferred from the angiotensin I (AngI) to AngII conversion. Incubation of patient and Ctr plasma with AngI demonstrated rapid conversion of AngI to AngII in the patient samples, supporting enhanced ACE activity (Figure 5I). While angiotensin‐converting enzyme 2 (ACE2) levels or activity could not be predicted from omics data, western blotting suggests potential ACE2 downregulation in patients.

### 2.6 Bioactive Peptides and Prediction of Novel Bioactive Peptides Associated with β‐Thalassemia

**Figure 6:**
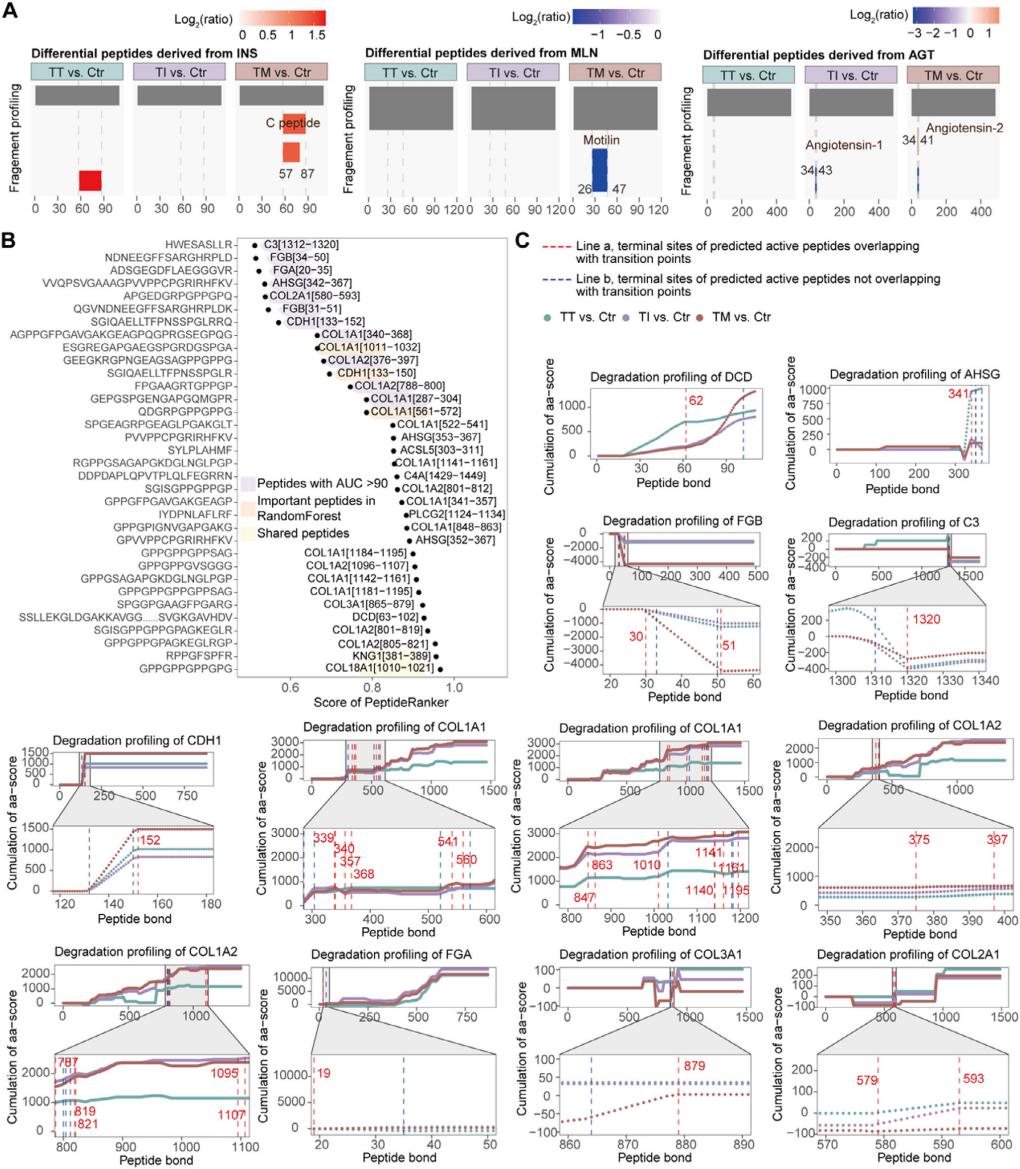
Prediction of bioactive peptides potentially associated with β‐thalassemia. A) Fragment profiling of proteins corresponding to known bioactive peptides identified in this study, including AngI, AngII, INS[57‐87] (C peptide), and MLN[26‐47] (motilin). B) Differential peptides analyzed by PeptideRanker; peptides with a score > 0.85 and potential biomarkers or key drivers of subgrouping with a score > 0.5 are shown. C) Cumulative aa‐score analysis highlighting protein regions with high likelihood of bioactivity, indicating potential bioactive peptides relevant to β‐thalassemia. The red numbers represent the bond positions indicated by Line a.

Proteolytic cleavage of precursor proteins to generate functional bioactive peptides represents a key mechanism for peptide production. Differential peptides were matched against known bioactive peptides. In addition to AngI and AngII, INS[57‐87] (C peptide) from insulin and MLN[26‐47] (motilin) from promotilin were identified (**Figure** **6**A). C peptide was significantly upregulated in TT and TM, whereas motilin was downregulated only in TM.

To identify novel bioactive peptides associated with β‐thalassemia, all differential peptides across the four groups were analyzed using PeptideRanker. Twenty peptides scored >0.85, and 18 additional peptides, identified as either potential biomarkers or key drivers of subgrouping, scored >0.5 (Figure 6B). Given the overlap between transition points and UniProt‐annotated functional sites, peptides with termini coinciding with transition points are more likely to possess functional relevance to β‐thalassemia (Figure 6C). Peptides with both termini overlapping transition points, such as COL1A1[1142‐1161], COL1A2[1096‐1107], COL1A1[848‐863], COL1A1[341‐357], COL1A1[1141‐1161], COL1A2[376‐397], COL1A1[340‐368], FGB[31‐51], and COL2A1[580‐593], as well as those with a single terminus overlapping important aa positions, including COL1A1[561‐572], COL1A2[788‐800], CDH1[133‐152], FGA[20‐35], and C3[1312‐1320]. May have high potential as bioactive peptides.

### 2.7 Extending Individual AA‐Score to the Discovery Stage of Biomarkers

**Figure 7:**
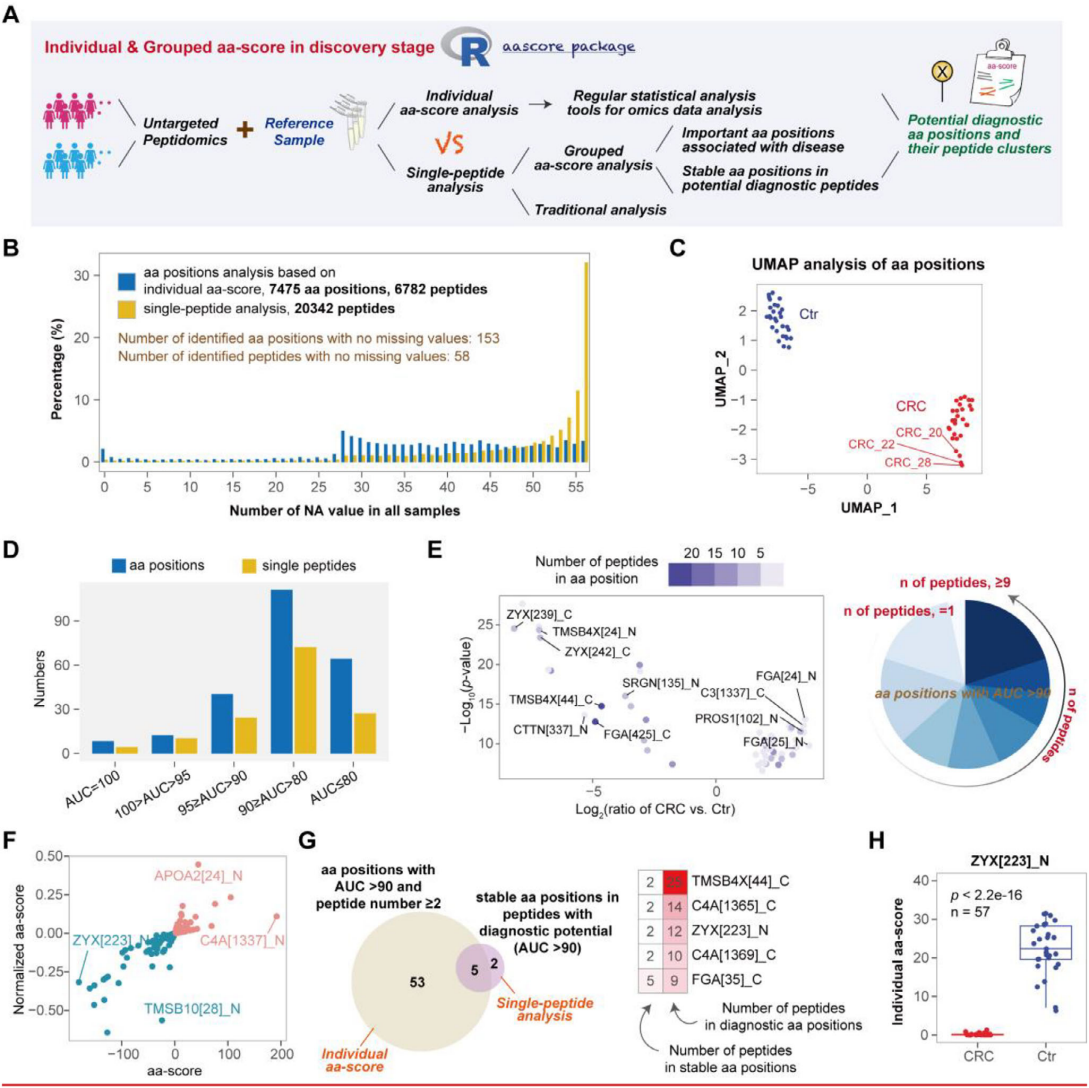
Application of individual aa‐score in a CRC cohort. A) Workflow illustrating the reference‐based aa‐score strategy. B) Distribution of missing values across the two analytical strategies. C) UMAP of aa positions quantified in ≥80% of samples per group. Each point represents an individual sample, colored by group (CRC in red, Ctr in blue). D) AUC distributions from ROC analyses, including only features quantified in ≥80% of samples per group. E) Volcano plot of aa positions with AUC > 0.9 supported by multiple peptides; adjacent pie chart shows proportional breakdown. F) Distribution of transition points identified via grouped aa‐score. G) Venn diagram showing overlap between stable points from diagnostically potent peptides and aa positions with AUC > 0.9 supported by multiple peptides; heatmap depicts peptide counts per aa position. H) Boxplot of individual aa‐score for ZYX[223]_N; median (centerline), upper/lower quartiles (box), and 1.5× IQR whiskers; significance assessed by Wilcoxon test; “n” indicates the number of samples.

As demonstrated in the PRM analyses above, the reference‐based individual aa‐score method is feasible and can be extended to the biomarker discovery stage. Compared with grouped aa‐scores, individual aa‐scores offer enhanced peptide coverage in reference samples through multiple technical replicates, reducing missing values and improving data utilization. To evaluate this, we performed plasma peptidomics on a cohort of 28 CRC patients and 29 Ctr, including five replicates of a reference sample (**Figure** **7**A). A total of 7205 peptides were quantified across these replicates.

Individual aa‐score analysis identified 7475 aa positions from 6782 peptides, whereas traditional single‐peptide analysis detected 20 342 peptides across 57 samples. Examination of missing values revealed that aa‐site‐based analysis substantially reduced data gaps (Figure 7B; Figure S7A, Supporting Information). Among the aa positions, 1766 sites, supported by 2327 peptides, were included in quantitative analysis, with 813 identified as differential. By comparison, conventional single‐peptide analysis quantified 1774 peptides, with 767 differential. Thus, reference‐based individual aa‐score analysis increased peptide utilization by 0.3‐fold and decreased missing values by 9.8%. These metrics could be further improved by diversifying or expanding reference samples. Analyzing aa positions based on reference also allows application of conventional omics statistical methods. Uniform Manifold Approximation and Projection (UMAP) dimensionality reduction analysis of aa positions demonstrated superior separation of CRC and Ctr groups compared with single‐peptide analysis (Figure 7C; Figure S7B, Supporting Information). ROC curve analyses further showed that aa positions exhibit greater diagnostic potential than single peptides (Figure 7D). The volcano plot (Figure 7E) highlights aa positions with an AUC >0.9, 96.7% of which were supported by two or more peptides.

Grouped aa‐score analysis of the same dataset identified 475 disease‐related transition points (Figure 7F). Cross‐examination of stable points from diagnostically potent peptides and diagnostically potent aa positions revealed five shared top candidates, including ZYX[223]_N, a disease‐related transition point (Figure 7G,H). To assess the influence of reference choice, we generated new reference samples by pooling equal volumes of CRC or Ctr plasma and re‐analyzed the cohort. Overlap analysis showed strong consistency between the original and new references: 71% of upregulated and 88% of downregulated aa positions were shared (Figure S7C, Supporting Information). Diagnostic aa positions (AUC > 0.9, ≥ 2 peptides) remained highly stable (Figure S7D,E, Supporting Information), with all top candidates retained across references (Figure S7F,G, Supporting Information). These results confirm that reference variability minimally affects the identification of differential or diagnostic aa positions, validating the robustness of this approach.

## 3 Discussion

Peptidomics remains underdeveloped due to analytical limitations. Conventional omics data analysis methods are ill‐suited for its unique challenges, particularly the redundancy arising from non‐specific enzymatic cleavage. While the clustering algorithm introduced by Hartman et al.^[^ ^28^ ^]^ represents progress, its clusters often lack clear biological interpretation, and parameter selection is manual, with thresholds unanchored in biological mechanisms. To address these limitations, we developed a single‐position peptide clustering strategy based on the aa‐score method, systematically evaluating proteolytic products generated under defined single‐substrate specificity and single‐cleavage‐site conditions, thereby establishing a biologically grounded analytical framework. This approach enabled the definition of a novel class of biomarkers—peptide cluster biomarkers based on protein aa site specificity—which integrate positional conservation and functional correlations derived from proteolytic signatures. Visualizations, such as aa‐score distributions and waterfall maps, map complex peptide changes back to their source proteins, facilitating multi‐group comparisons and the effective extraction of meaningful peptide information. These computational methods have been implemented in an R package to enhance accessibility and user‐friendliness. Application to a β‐thalassemia cohort revealed novel insights.

To optimize peptidomics workflows, especially for challenging plasma samples, we employed our previously developed high‐efficiency plasma peptide extraction method, SPD. This method enhances identification confidence and depth, while maintaining reproducibility and quantitative stability,^[^ ^17^ ^]^ making it suitable for clinical research. Protein identification, supported by multiple tryptic peptides, generally exhibits higher reliability than single peptides under multiple FDR controls. Consequently, peptidomics research must carefully consider peptide identification reliability. Building on strategies from microprotein identification,^[^ ^29^ ^]^ we rigorously defined endogenous peptides in this study.

The plasma peptide profiles of β‐thalassemia subgroups (TT, TI, and TM) remain poorly characterized. Using label‐free quantitative LC‐MS/MS, we delineated subgroup peptidomic patterns. Notably, aa‐score‐based analysis of AHSG degradation more accurately reflected in vivo enzymatic activity, whereas conventional methods could yield misleading conclusions. Integrating traditional differential peptide analysis with aa‐score profiling, we identified aa positions most strongly associated with β‐thalassemia, including upregulated sites SRGN[130]_N, APOC3[21]_N, SRGN[89]_C, and PLXDC2[90]_C, and downregulated sites IGF2[126]_C, FGB[31]_N, ITIH4[688]_C, and APOA4[304]_C. These critical positions often correspond to peptide endpoints with discriminative power or to functionally annotated sites in UniProt. For instance, PLXDC2[71‐90] and PLXDC2[76‐90] represent top potential screening biomarkers, with multiple peptides sharing the same C‐terminal showing significant differences. Similarly, apolipoprotein C‐III (APOC3) fragments [21‐39], [21‐42], and [21‐43] (AUCs > 90%) consistently highlight site [21], the N‐terminal of APOC3 chain. Well‐studied positions such as ITIH4[688]_C, FGB[31]_N, and IGF2[126]_C were confirmed, while SRGN fragments were uniformly upregulated, indicating potential for disease screening.

In subsequent targeted quantitative analysis, we deviated from conventional approaches by incorporating heavy‐labeled peptides and reference samples to calculate individual aa‐scores, validating the differential nature of these key positions. Individual aa‐scores identified PLXDC2[90]_C, CDH1[152]_C, and IGF[126]_C as stable diagnostic positions, informing an implementation strategy that utilized these sites for screening. Although multiple individual aa‐score calculation strategies proved robust across PRM batches, absolute quantification is recommended. Heavy‐labeled peptides address the missing‐value challenges intrinsic to peptidomics and enhance the reproducibility and stability of diagnostic analyses. References can further improve peptide cluster selection success rates while reducing research costs.

In addition, predicted protease analyses and cognate precursor protein profiling revealed several signaling pathways implicated in β‐thalassemia. Proteins associated with apoptosis and lysosomal pathways yielded more peptides, whereas proteins within the RAS pathway produced fewer. Apoptotic and lysosomal abnormalities have been consistently observed in β‐thalassemia plasma proteomics, yet cathepsins were undetected, highlighting how peptidomics complements proteomic insights. Lysosomal dysfunction induced by HSP90 and subsequent ferroptosis events likely contribute to disease pathology, though the precise cellular origins remain unclear due to the heterogeneous sources of plasma proteins and peptides. These findings align with prior reports of accelerated apoptosis in erythroid precursors.^[^ ^30^ ^]^

The RAS pathway plays a central role in extracellular volume homeostasis and blood pressure regulation. REN mediates AngI generation from AGT, and ACE converts AngI to AngII. Plasma proteomics revealed no changes in AGT or ACE abundance in β‐thalassemia patients, suggesting that reduced REN activity could decrease AngI production. Peptidomic analysis additionally indicated diminished MME activity, limiting AngI‐to‐Ang1‐7 conversion. Notably, both non‐targeted and targeted peptidomics detected increased AngII but not Ang1‐7, precluding direct inference of ACE2 activity. Western blotting, however, revealed downregulation of ACE2 in patients, potentially explaining observations by Haghpanah et al.^[^ ^31^ ^]^ of lower COVID‐19 incidence in β‐thalassemia, with emerging evidence suggesting partial protective immunity against SARS‐CoV‐2^[^ ^32^, ^33^ ^]^ linked to reduced ACE2 expression.

AngII also interacts with MGP, a key regulator of vascular calcification. Fragment analysis of MGP revealed upregulation of MGP[20‐36] and downregulation of MGP[30‐47], complicating interpretation; however, aa‐score profiling indicated enhanced MGP degradation in patients, which may exacerbate pathological calcification. Furthermore, osteoporosis and osteopenia are common in β‐thalassemia. Type I collagen, encoded by the COL1A1 and COL1A2, is the main structural protein in bone, and both proteins showed significant degradation in patient plasma. Given MGP's role in promoting bone formation,^[^ ^34^ ^]^ its degradation alongside type I collagen may contribute to skeletal fragility in this population.

Collectively, these results underscore the methodological advantage of the aa‐score‐driven strategy and support integrating next‐generation peptidomics with proteomics and complementary technologies for comprehensive mechanistic studies. Application to a CRC cohort further refined the aa‐score framework, demonstrating its clinical utility and generalizability across disease contexts. Comparative evaluation against conventional peptide profiling confirmed that, while reference pools remain necessary, the individual aa‐score approach offers distinct benefits in managing missing values and identifying diagnostically relevant aa positions. The minimal impact of reference variability on analytical outcomes reinforces its suitability for initial biomarker discovery. Importantly, identified aa positions require systematic validation using heavy‐labeled peptide‐based absolute quantification to ensure reproducibility and facilitate clinical translation. Overall, this end‐to‐end, aa‐score‐enabled peptidomic workflow establishes a robust platform for mapping disease‐associated proteolytic landscapes and advancing peptide cluster‐based diagnostics through functionally annotated molecular profiling.

## 4 Experimental Section

### Clinical Sample Collection

In the discovery phase of peptidomics, 8 individuals with TT (β^+^β^N^/β^0^β^N^, 10.1 ± 3.9 years), 17 patients with TI (β^+^β^+^/β^+^β^0^, 9.1 ± 2.8 years), 16 patients with TM (β^0^β^0^, 8.8 ± 2.2 years), and 13 healthy controls (9.8 ± 1.7 years) were enrolled, matched for age and gender. In the subsequent targeted quantitative peptidomics (PRM), Batch 1 included 16 individuals with TT (β^0^β^N^, 34.4 ± 3.4 years), 17 patients with TM (β^0^β^0^/β^0^β^+^, 10.6 ± 2.1 years), and 17 healthy controls (32.9 ± 3.7 years). Batch 2 comprised 39 individuals with TT (β^0^β^N^/β^+^β^N^, 38.2 ± 8.2 years), 12 patients with TM (β^0^β^0^/β^0^β^+^, 9.5 ± 2.9 years), and 40 healthy controls (37.7 ± 12.5 years). One control in Batch 1 was excluded due to aberrant peptide quantifications during quality control. Peripheral blood was collected at the First Affiliated Hospital of Guangxi Medical University (Guangxi Zhuang Autonomous Region, China), with study approval from the institutional Ethics Committee. Written informed consent was obtained from patients or their legal guardians. β‐thalassemia genotyping followed previously described protocols,^[^ ^14^ ^]^ and none of the participants had undergone splenectomy. Plasma was isolated from EDTA‐anticoagulated blood by centrifugation at 3000 × *g* for 10 min and stored at −80 °C. For transfusion‐dependent patients, blood was collected 2 weeks post‐transfusion to minimize external influences.

A CRC cohort, comprising 28 patients (54.9 ± 10.6 years) and 29 age‐ and gender‐matched healthy controls (49.5 ± 7.9 years), was recruited from the First Affiliated Hospital of Henan University (Kaifeng, China), with all procedures approved by the Ethics Committee of Biomedical Science and Research, Henan University. Written informed consent was obtained from all participants. CRC diagnosis followed a multidisciplinary protocol integrating CT imaging, endoscopic histopathology, and clinical assessments, consistent with the 2023 NCCN Clinical Practice Guidelines. The original reference sample was prepared by pooling equal volumes of all plasma samples from the CRC cohort. The new reference samples were generated by separately pooling equal volumes of CRC and Ctr plasma.

### Enrichment of Plasma Peptidome

Reference plasma was prepared by pooling equal volumes of individual samples. Peptides were enriched using the SPD method.^[^ ^17^ ^]^ Briefly, 50 µL plasma was mixed with 50 µL deionized water and 250 µL methyl‐*tert*‐butyl ether (MTBE). After vortexing, 150 µL of methanol was added to precipitate proteins. Samples were incubated at 4 °C for 30 min and centrifuged at 21 000 × *g* for 30 min at 4 °C. The supernatant was transferred to a new tube. For delipidation, 500 µL of MTBE and 100 µL of deionized water were added, centrifuged at 1000 × *g* for 10 min at 4 °C, and the upper phase was discarded. The lower phase was lyophilized under vacuum at 4 °C.

For CRC samples, peptide enrichment followed a modified SPD protocol. Plasma was reduced with 20 mm dithiothreitol (DTT, Sigma–Aldrich) for 1 h at 4 °C and then alkylated with 40 mM Iodoacetamide (IAM, Sigma–Aldrich) at 4 °C in the dark for 30 min. Post‐SPD processing, lipid interference was minimized by secondary delipidation with 400 µL of the upper phase of an MTBE/MeOH/H_2_O (5:1:1, v/v) system.

Samples were desalted using the EasyPept 96‐well plate system (Omicsolution, China). Dried peptides were dissolved in 150 µL of 0.1% trifluoroacetic acid (TFA) and then loaded onto desalting columns twice to ensure thorough binding of the peptides to the resin. Centrifugation at 700 × *g* for 1 min was used to drive the sample through the resin and remove unbound contaminants. Columns were washed twice with 100 µL D buffer and twice with 100 µL E buffer. Peptides were eluted using 40 µL of F buffer, repeated three times for a total volume of 120 µL, and dried under vacuum centrifugation at 4 °C.

### Non‐Targeted Peptidomics Using LC‐MS/MS

NanoLC‐MS/MS analyses were performed on an Orbitrap Exploris 480 mass spectrometer (Thermo Scientific) with FAIMS Pro interface, coupled to an Easy n‐LC 1200 HPLC system (Thermo Scientific). Desalted peptides were resuspended in 0.1% FA and loaded onto a C18 trap column (100 µm × 2 cm, Reprosil‐Pur C18AQ, 5 µm, Dr. Maisch GmbH, Germany) and separated on a C18 capillary column (75 µm × 20 cm, Reprosil‐Pur C18 AQ, 3 µm, Dr. Maisch GmbH, Germany). Peptides were eluted with a 103‐min linear gradient using mobile phase A (0.1% FA) and B (ACN/0.1% FA): 4–11% B, 4 min; 11–21% B, 28 min; 21–30% B, 29 min; 30–42% B, 27 min; 42–95% B, 5 min; 95% B, 10 min at a flow rate of 300 nL min^−1^. FAIMS separations used compensation voltages of −45 and −65 V. In data‐dependent acquisition (DDA) mode, full MS scans were acquired at a resolution of 60 000 (at m z^−1^ 200) across a mass range of 350–1600 m z^−1^, with a normalized AGC target of 300% and a maximum injection time of 80 ms. Precursor ions were isolated with a 1.6 m z^−1^ precursor isolation width and fragmented via HCD at 28% collision energy. MS/MS spectra were acquired at a resolution of 60 000 (at m z^−1^ 200) with a normalized AGC target of 150% and a maximum injection time of 118 ms. The dynamic exclusion duration of ions for MS/MS was set to 30 s.

Non‐targeted peptidomics raw data were initially processed using pFind, followed by Proteome Discoverer 2.4 with the Sequest HT search engine against the UniProt human protein database (downloaded 09/2021). Key search parameters were configured as follows: no enzyme; precursor tolerance, 10 ppm; fragment tolerance, 0.02 Da. Variable modifications included oxidation of methionine and proline, cysteinyl modification of cysteine, and N‐terminal conversion of glutamine to pyroglutamate. Peptide‐level FDR was controlled at ≤1% using Percolator. To correct for sample loading variability, peptide abundances were normalized by total peptide amount and scaled to 100 in Proteome Discoverer 2.4, yielding the scaled abundances used in subsequent analyses. Only peptides with spectra containing at least four continuous b‐ or y‐ions were retained for downstream analyses to enhance identification confidence.

### Spectral Library Construction and PRM Analysis

PRM‐MS/MS analyses were conducted on an Orbitrap Eclipse Tribrid mass spectrometer coupled to an EASY‐nLC 1200 HPLC system, as previously described.^[^ ^35^ ^]^ Peptides were separated using mobile phase A (0.1% FA) and B (ACN/0.1% FA) with a 73‐min linear gradient: 5–10% B, 3 min; 10–20% B, 22 min; 20–30% B, 22 min; 30–40% B, 13 min; 40–99% B, 4 min; 95% B, 9 min at a flow rate of 300 nL min^−1^. Samples were spiked with 10 × iRT standard peptides (Biognosys) for retention time (RT) calibration. Full MS scans were acquired at a resolution of 60 000 at m z^−1^ 200 across the mass range of 350–1200 m z^−1^ with an AGC of 6e5 and maximum injection time of 50 ms. MS/MS spectra were acquired at a resolution of 30 000 at m z^−1^ 200, an isolation window of 1 m/z, a HCD collision energy of 30% and an AGC target value of 2e5 with a maximum injection time of 80 ms.

PRM analyses were performed using SpectroDive 12.1 (Biognosys). Spectral libraries were generated by searching DDA raw data with the Pulsar engine against the human SwissProt FASTA database (20601 entries). Initial unscheduled PRM experiments on three randomly selected samples per group were used to identify precursors suitable for monitoring and for guiding heavy‐labeled peptide synthesis. DDA data were integrated with the first‐round unscheduled PRM data to construct the final spectral library. A spike‐in panel of heavy‐labeled peptides was developed based on this library. For method optimization, 20 µL of plasma from each sample in the Batch 1 PRM cohort was pooled to create mixture samples. After mixing the heavy‐labeled peptides with the mixture sample, a second round of unscheduled PRM was performed to optimize precursor selection and RT calibration. For absolute quantification, heavy‐labeled peptides were mixed in proportion to endogenous peptides in the mixture sample, followed by gradient dilution to prepare eight mixture samples for the construction of a standard curve. Scheduled PRM data acquisition was then performed for each sample with a ±2.5 min RT window.

Search parameters for spectral library generation were set as follows: no enzyme, unspecific digestion, maximum peptide length 40, variable modifications including oxidation of methionine and proline, and N‐terminal glutamine conversion to pyro‐glutamate (Gln→pyro‐Glu[AnyN‐termQ]); all other settings were default. The targeted quantitative analysis included: individual peptides with diagnostic potential, representative peptides corresponding to aa positions of diagnostic significance, non‐modified and modified forms of AHSG[312‐339] and HAMP[25‐48], and AngI/AngII. Several key parameters for panel generation were set as follows: precursor m/z, 350–1500; precursor charge, 2–6; peptide length, 7–40; proteotypicity, all peptides; allowed modification, carbamidomethyl (C); fragment m/z, 300–1800; max fragment charge, 3; Ion types, b and y; allowed loss types, H_2_O, NH_3_ and noloss.

Precursors or fragments with poor signal and peak shape were discarded. For PRM data analysis, a q‐value cutoff of 0.01 was set for peptide selection. All selected peaks were manually checked after automated peak detection by SpectroDive. Product ion signal peaks with significant interfering signal around the peak apex were excluded, and at least three transitions per precursor were selected for quantification. During manual review, the absence of a heavy‐labeled peptide within a peptide cluster due to RT shift indicated the corresponding endogenous peptide was undetected, and the sample was excluded from aa position calculations.

### Targeted Position and Peptide Selection

Biomarker candidate positions were selected based on dual criteria: i) significant diagnostic potential at the positional level, and ii) presence of ≥2 individual peptide within the corresponding peptide cluster demonstrating independent diagnostic capacity. These positions were prioritized in untargeted peptidomics for subsequent targeted PRM validation. This strategy enables maximized representation of diagnostically relevant peptide clusters using minimal signature peptides and resource‐efficient biomarker development through focused analysis of high‐value targets.

For the initial unscheduled PRM experiments, the selection principle prioritized maximal peptide inclusion per position. Subsequently, peptide retention was conditional upon: i) PRM targetability, ii) high signal intensity, iii) well‐resolved chromatographic peaks without co‐eluting interference, and iv) high AUC values. Synthesis prioritized peptides with the highest AUC values. Although comprehensive synthesis remained technically feasible, the optimization strategy integrated economic constraints with position‐specific biological relevance. The synthesis strategy followed a tiered approach. At positions containing dominant peptides (AUC = 100%), 2–4 peptides were synthesized, ensuring inclusion of all 100%‐AUC candidates; for positions lacking dominant peptides (all candidates < 100% AUC), additional peptides were synthesized to ensure comprehensive coverage.

### Data and Bioinformatic Analysis

Tissue Cell type and term enrichment heatmap were generated using Metascape (version 3.5), and KEGG and protein‐protein interaction analysis were performed with STRING (version 11.5). Protease activity was predicted using Proteasix,^[^ ^36^ ^]^ quantified by normalizing the number of peptides cleaved by each predicted protease to the total number of cleavage sequences across all predicted proteases, multiplied by 100%. Data analysis was performed using R (version 4.2.2) in RStudio. The receiver operating characteristic (ROC) curve analysis was conducted with the pROC package (version 1.18.5), and UMAP analysis was performed using the uwot package (version 0.1.16). For both ROC and UMAP analyses, only features with a missing value rate of less than 20% in each group were included. Heatmap clustering was performed using R package pheatmap (version 1.0.12). R package corrplot (version 0.94) was used for correlation analysis. Key peptides for subgroup classification were identified using a random forest model (R package randomForest, version 4.7‐1.1). R package ggplot2 (version 3.5.1) was used for data visualization.

The aascore R package (version 1.1.0) was implemented to calculate aa‐scores, identify changing, stable, and transition points, determine key dysregulated aa positions, and generate graphical outputs tailored to these analyses. Transition points on waterfall maps were defined as changing points with positive, negative, or zero changes in slope, reflecting the most pronounced variations in measured variable.

### Peptide Bond Position Specification and Calculation of Grouped AA‐Score

For a protein sequence comprising *L* amino acids, peptide bond positions are indexed from 0 to *L* as follows:

1. Position 0: Virtual bond preceding the N‐terminal amino acid (aa_1_)
2. Position *j* (1 ≤ *j* ≤ *L*−1): Actual peptide bond between aa*_j_* and aa*_j_*_+1_
3. Position *L*: Virtual bond following the C‐terminal amino acid (aa*_L_*)

Points were defined as either aa positions or peptide bond positions. The peptide bond position at the N‐terminus of a peptide corresponds to the aa position minus one, while at the C‐terminus it equals the aa position. The position orientation (_N or _C) indicates the terminal exposure of detected proteolytic products. The “_B” suffix designates sites where proteolytic products from both orientations co‐exist.

The grouped aa‐score quantifies localized differential signals across multiple peptides at specific proteolytic sites. Specifically, when comparing two groups (e.g., Group 1 vs Group 2), the fold change for a given peptide was always calculated by comparing the signal intensity of the peptide in the higher‐intensity group to that in the lower‐intensity group. This ensures consistency in the direction of the comparison.

1. When the peptide signal in Group 1 was higher than that in Group 2, the fold change was positive, indicating an increase in grouped aa‐score for that peptide bond position.
2. When the peptide signal in Group 1 was lower than that in Group 2, the fold change was negative, indicating a decrease in grouped aa‐score for that peptide bond position.

This approach ensures that the score reflects the relative changes in peptide intensity between the two groups, rather than being dependent on the absolute intensity values of the peptides themselves. Subsequently, the fold change value of each peptide was assigned to all peptide bonds within that peptide. The grouped aa‐score for a given peptide bond position was then calculated as the sum of fold change values from all differential peptides spanning that specific bond position.

The grouped aa‐score at position *j* is calculated as:

(1) $$\mathrm{Grouped}\;\mathrm{aa}-\mathrm{score}\;\left(j\right)=\sum_{k=1}^{n_{j}}fc_{k}$$

where:

1. *j* = Peptide bond position index (0 ≤ *j* ≤ *L*)
2. *n_j_* = Total number of differential peptides spanning position *j*
3. *fc_k_* = Signed fold change of the *k*‐th peptide spanning position *j*

The number of peptide bonds per protein (*L*‐1) was used to normalize grouped aa‐scores or cumulative grouped aa‐scores.

### Statistical Analysis

All statistical analyses were performed using R (version 4.2.2) in RStudio. Within each group, peptides or aa positions identified or quantified in at least three samples were included for qualitative or quantitative analyses, respectively. Statistical comparisons were performed using two‐sided two‐sample *t*‐tests on log_2_‐transformed abundance of peptide signatures and aa positions. Features with *p*‐value < 0.05 and fold change > 2 or < 0.5 were considered upregulated or downregulated. For specific peptides or aa positions shown in boxplots, two‐group comparisons were performed using two‐sided Wilcox tests. Three‐ or four‐group comparisons were analyzed via one‐way ANOVA. Statistical significance was defined as *p* < 0.05 (*p < 0.05, **p < 0.01, ***p < 0.001). Boxplots depict the median (centerline), upper and lower quartiles (box limits), and whiskers spanning 1.5× IQR. “*n*” indicates the number of samples per boxplot.

### Peptide Synthesis

Peptides were synthesized by SynPeptide (Nanjing, China) via standard Fmoc solid‐phase peptide synthesis on 2‐chlorotrityl chloride resin. Sequential amino acid coupling, Fmoc deprotection with 20% piperidine in DMF, and final TFA‐based cleavage from resin were performed. Crude peptides were purified by HPLC and lyophilized to yield the final products.

### Western Blot Analysis

Plasma samples from Ctr, TI, and TM groups were analyzed to quantify AGT (1:1000, ab276132, Abcam), Renin (1:1000, ab125012, Abcam), MME (1:1000, ab256494, Abcam), ACE (1:1000, ab254222, Abcam), ACE2 (1:1000, ab108252, Abcam) and AHSG (1:1000, ab137125, Abcam) following standard protocols. Protein concentrations were measured with the Pierce BCA kit (23227, Thermo Scientific), and 10 µg of total protein was loaded per lane.

### Matrix‐Assisted Laser Desorption/Ionization Time‐of‐Flight Mass Spectrometry Analysis

To assess AngI‐substrate enzyme activity in β‐thalassemia, plasma from three TM patients and three Ctr volunteers was incubated with synthetic AngI peptide. A total of 10 µL of plasma was incubated with 0.1 µg AngI peptide for 6 h, with sampling every 2 h. For each time point, 1 µL of the reaction was mixed with 10 µL of 0.1% FA, desalted using a C18 ZipTip, vacuum‐dried at 4 °C, and then reconstituted in 5 µL of 0.1% FA. Subsequently, 1 µL of reconstituted sample was mixed with 1 µL internal standard peptide (EIVLTQSPDTLSLSPGER, 3.3 ng µL^−1^) and 1 µL CHCA matrix (5 µg µL^−1^), then directly spotted onto the MALDI target plate for analysis using MALDI‐TOF MS.

## Conflict of Interest

The authors declare no conflict of interest.

## Author Contributions

N.L., Y.Z., Y.Y., and J.W. contributed equally to this study. F.Y., N.L., and X.G. supervised the study and reviewed the manuscript. J.L., A.P., and X.G. collected clinical samples. N.L. developed the aa‐score algorithm, analyzed the data, wrote the R package aa‐score (1.1.0), and drafted the manuscript. N.L., Y.Z., and Y.Y. performed the experiments. Y.Y. assisted with data analysis. J.W. collected the LC‐MS/MS data. L.N., X.D., M.Z., Z.X., T.C., and X.G. assisted in project supervision. All authors approved the final version of the manuscript.

## Supporting information



Supporting Information

Supplemental Date File

## Data Availability

The data that support the findings of this study are openly available in [ProteomeXchange Consortium] deposited via the iProX partner repository at [https://www.iprox.cn/page/PSV023.html;?url = 1748240604657FClz], reference number [PXD062496].
