# Supplementary material for: Single‐Position Peptide Clustering for Peptidomics Reveals Novel Disease Biomarkers and Dysregulated Proteolytic Characteristics
Source: Adv Sci (Weinh). 2025 Nov 18;13(5):e10910. doi: 10.1002/advs.202510910 (PMC12850352; doi:10.1002/advs.202510910)

**Supplementary Information**

**Single-Position Peptide Clustering for Peptidomics Reveals Novel Disease Biomarkers and Dysregulated Proteolytic Characteristics**

Na Li^1#^*, Yaxin Zhu^1#^, Yumeng Yan^1#^, Jifeng Wang^1#^, Lili Niu^1^, Xiang Ding^1,2^, Mengmeng Zhang^1^, Zhensheng Xie^1,2^, Tanxi Cai^1,2^, Xiaojing Guo^1^, Jianming Luo^3^, Peng An^4^, Xiangqian Guo^5^*, Fuquan Yang^1,2^*

^1^Laboratory of Proteomics, Institute of Biophysics, Chinese Academy of Sciences, Beijing 100101, China;

^2^University of Chinese Academy of Sciences, Beijing 100049, China;

^3^Department of Pediatrics, The First Affiliated Hospital of Guangxi Medical University, Nanning 530021, China;

^4^Department of Nutrition and Health, China Agricultural University, Beijing 100193, China;

^5^Henan Provincial Engineering Center for Tumor Molecular Medicine, Zhongyuan Intelligent Medical Laboratory, School of Basic Medical Sciences, Henan University, Kaifeng 475004, China.

**^#^ These authors contributed equally to this study.**

*** Correspondence**

Fuquan Yang, Laboratory of Proteomics, Institute of Biophysics, Chinese Academy of Sciences, Beijing 100101, China; or University of Chinese Academy of Sciences, Beijing 100049, China. Email: [fqyang@ibp.ac.cn](mailto:fqyang@ibp.ac.cn).

Na Li, Laboratory of Proteomics, Institute of Biophysics, Chinese Academy of Sciences, Beijing 100101, China. Email: [lina@ibp.ac.cn](mailto:lina@ibp.ac.cn).

Xiangqian Guo, Henan Provincial Engineering Center for Tumor Molecular Medicine, Zhongyuan Intelligent Medical Laboratory, School of Basic Medical Sciences, Henan University, Kaifeng 475004, China. Email: [xqguo@henu.edu.cn](mailto:xqguo@henu.edu.cn).

**Supplementary Data**

**Supplementary Data 1:** Identified peptides in each group.

**Supplementary Data 2:** Differential peptides.

**Supplementary Data 3:** Result of ROC analysis.

**Supplementary Data 4:** Result of Batch 1 PRM analysis.

**Supplementary Data 5:** Result of Batch 2 PRM analysis.

**Supplementary Figures**

**Figure S1. Quality of MS/MS spectra and results of peptide identification.**

A) Poor-quality MS/MS Spectra with FDR < 0.05.

B) Identification frequency of peptides in each group.

C) Tissue- and cell-specific enrichment analysis of proteins corresponding to the identified peptides in each group.


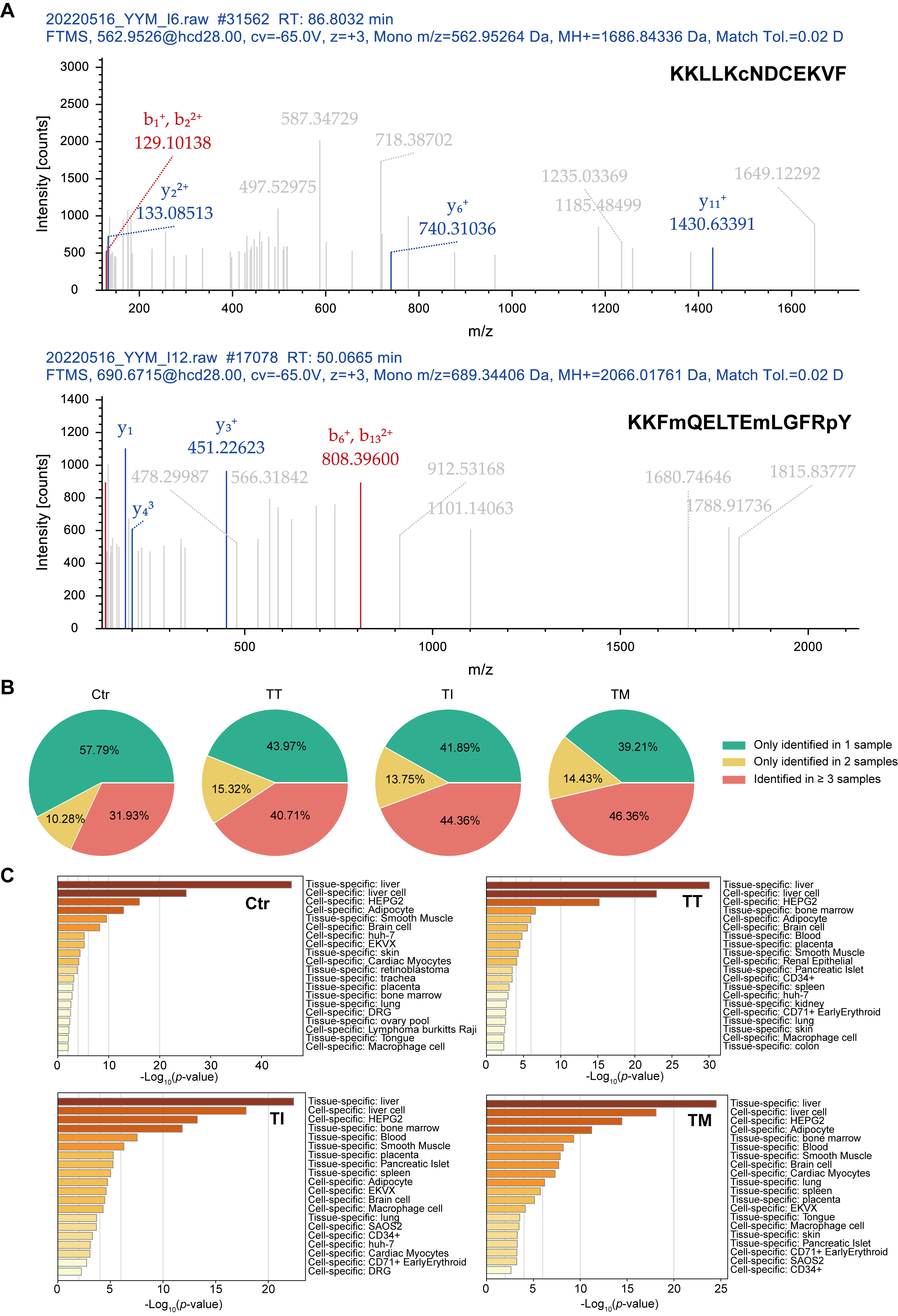


**Figure S2. Fragment profiling of proteins.**

A) Differential peptides derived from TF, HBA1, and HBB.

B) Identified unique peptides derived from TF, HBA1, HBB, MPO, FTL, and HPX.


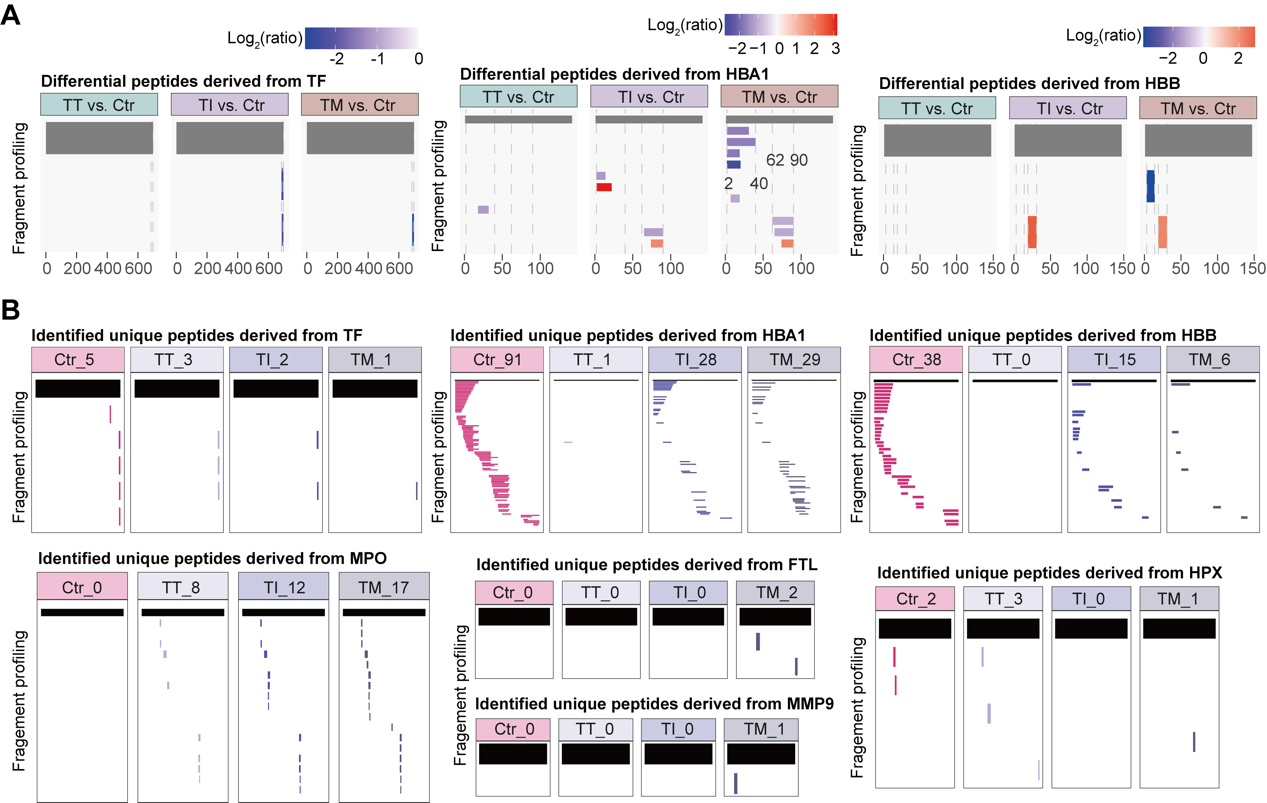


**Figure S3. Analysis of the diagnostic potential of peptide clusters and single peptides.**

ROC (A) and boxplot (B) analyses of CDH1[152]_C, PLXDC2[90]_C, SRGN[130]_N, AHSG[339]_C, C3[1320]_C, and SRGN[72]_N using individual aa-score based on reference sample with only target precursors.

C) Boxplot showing the individual aa-score of aa positions based on reference with all precursors.

D) ROC and boxplot analyses of single precursors involved in the calculation of aa positions in PRM analysis. Comparisons between the β-thalassemia and Ctr were analyzed using the non-parametric Wilcoxon rank-sum test. The centerline in the boxplot depicts the median, the box limits depict the upper and lower quartiles, and the whiskers encompass 1.5 times the interquartile range (IQR). "n" indicates the number of samples.


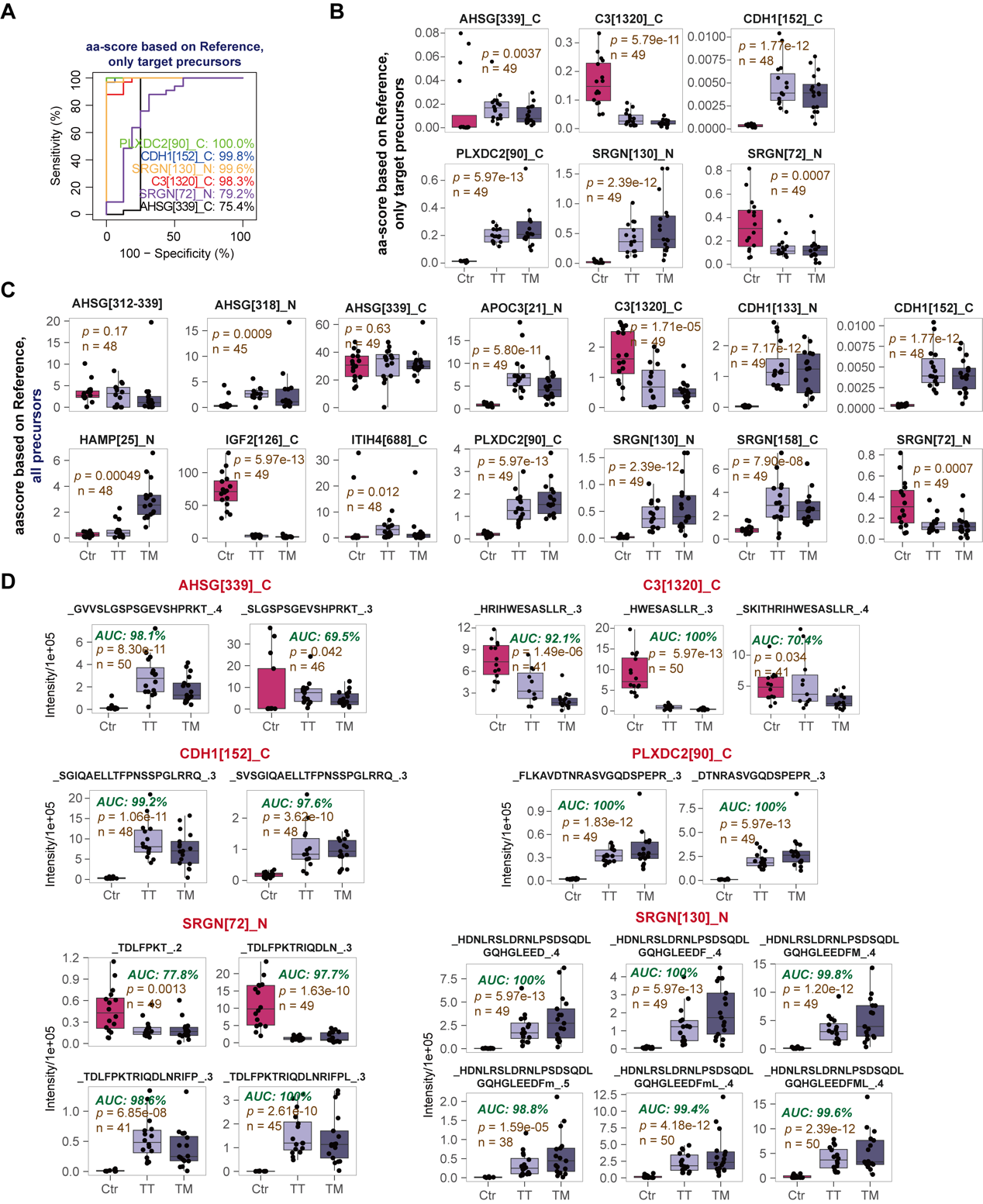


**Figure S4. The result of PRM analysis.**

Representative peak group chromatograms of precursors derived from PLXDC2[90]_C (A), CDH1[152]_C (B), and IGF2[126]_C (C).

D) Boxplot of the individual aa-score of PLXDC2[90]_C and IGF2[126]_C in Batch 2 of PRM.

E) ROC analysis of combined PRM data from both batches.

F) The scaled abundance of PLXDC2, IGF2, and CDH1 quantified using plasma proteomics.


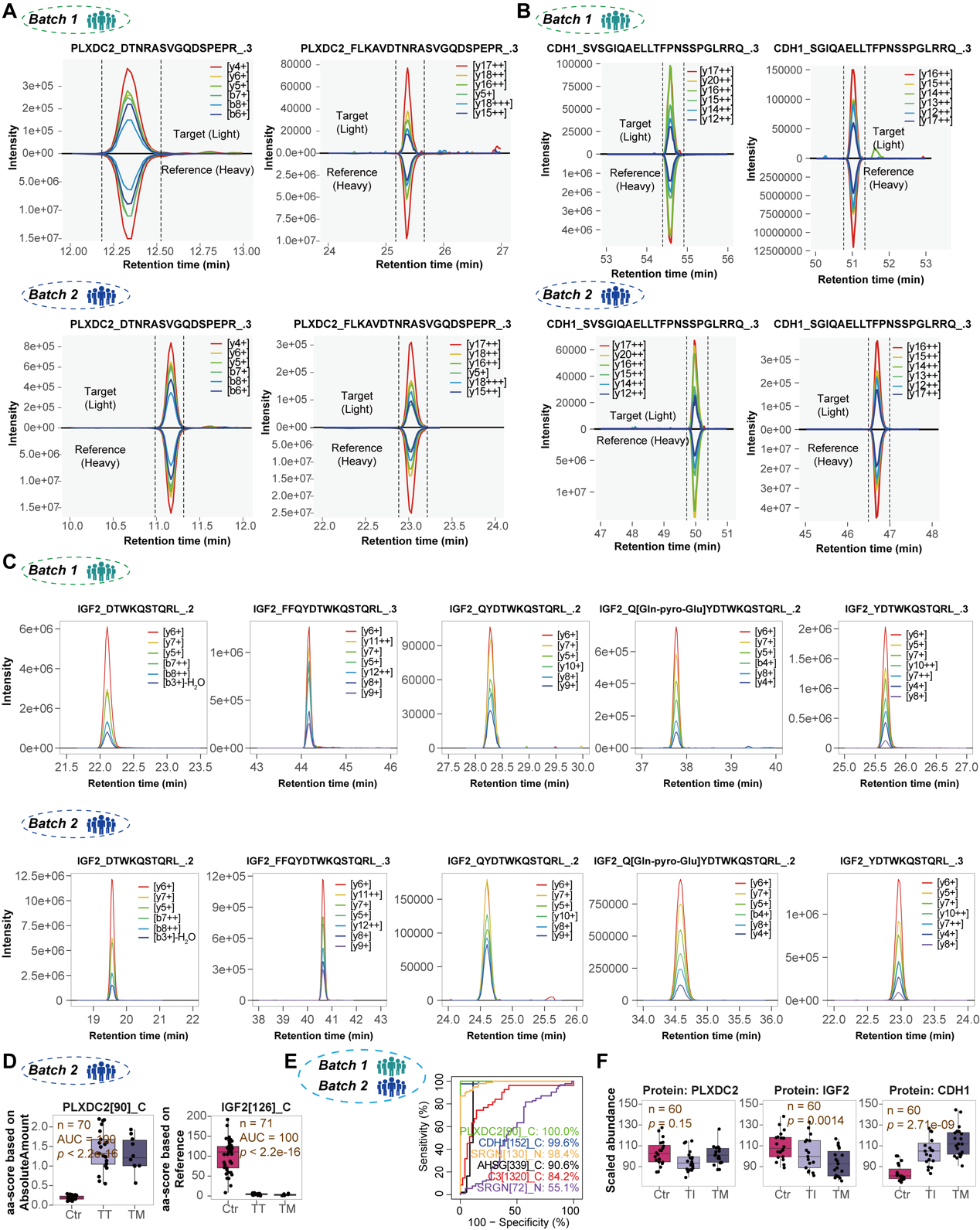


**Figure S5. Pearson correlation analysis.**

A) Pearson correlation matrix of the top 20 peptides, revealing significant correlation patterns.

B) Pearson correlation analysis of IGF2[116-126] and ZYX[223-237]. "n" indicates the number of samples.

**
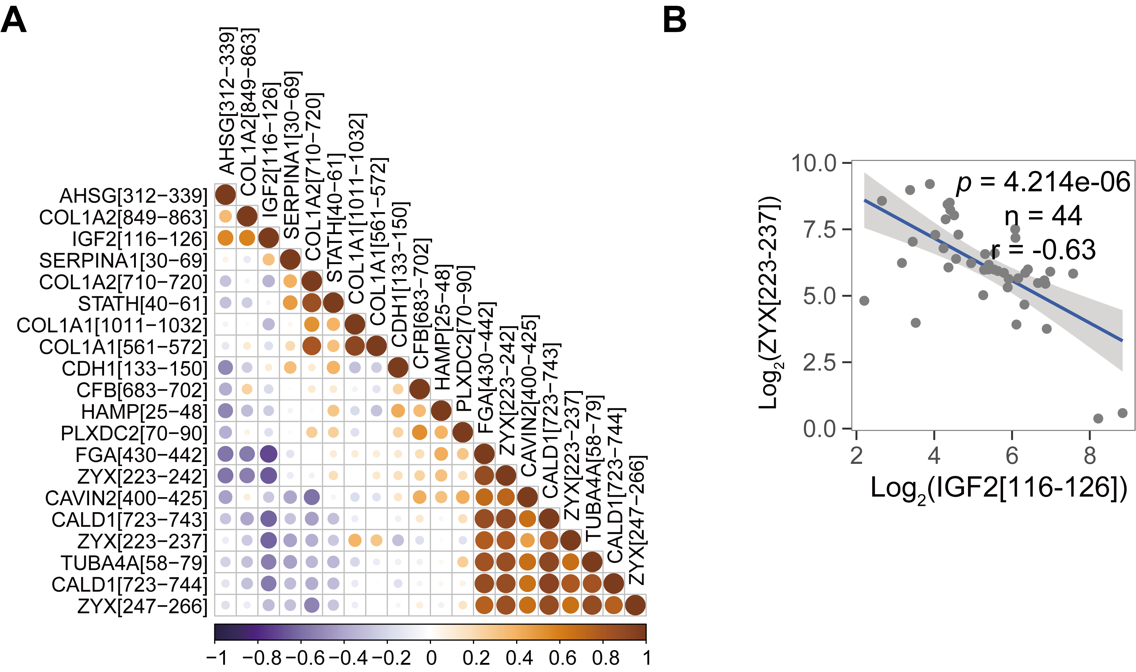
**

**Figure S6. Analysis of predicted proteases and pathway-associated plasma proteins.**

A) Heatmap analysis of predicted proteases.

Enrichment analyses of activated or inhibited proteases using Metascape (B) and STRING (C).

D) Quantitative analysis of HSP90 isoforms (HSP90AA1 and HSP90B1), LAMP-2a (LAMP2), HSC70 (HSPA8), and lysosomal hydrolases/cofactors in our plasma proteomics study. Sample size n was 20 per group.

E) The increased cytochrome c (CYCS) levels in our plasma proteomics study. Sample size n was 20 per group.

F) Boxplot of proteases involved in apoptosis pathway. The centerline in the boxplot depicts the median, the box limits depict the upper and lower quartiles, and the whiskers encompass 1.5 times the interquartile range (IQR). Sample size n was 53.

G) Integrated proteomic and peptidomic analysis of β-thalassemia plasma reveals enhanced ferroptosis and apoptotic signaling pathways. Key upregulated proteins identified in this study are highlighted in red.

H) Grouped aa-score analysis of MGP.


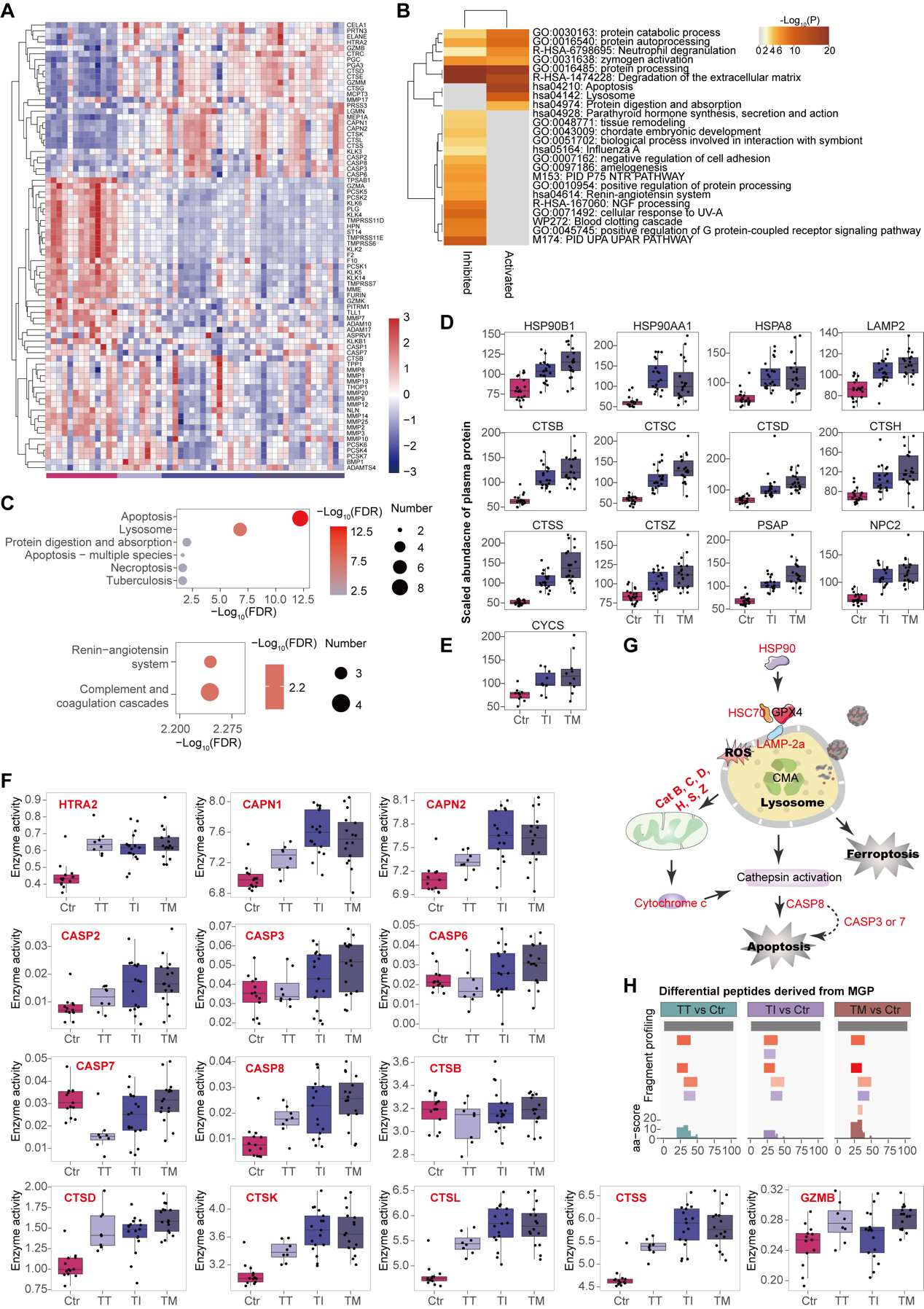


**Figure S7. Analysis of aa positions or single peptides participating in differential analysis.**

A) Missing value distribution in the quantified features participating in differential analysis across two analysis strategies.

B) UMAP analysis of single peptides with quantitative differences within 80% of samples in each group. Each point represents an individual sample, colored by group (CRC in red, Ctr in blue).

C) Overlap analysis of differential aa positions between the original and new references.

D) Overlap analysis of aa positions with diagnostic potential between the original and new references.

E) The AUC distribution of aa positions where the individual aa-score is calculated using the new reference.

F) The Venn diagram shows the shared sites of stable points from peptides with diagnostic potential and aa positions with an AUC greater than 0.9 and more than one peptide (calculated using the new reference). The heatmap displays the number of peptides involving in each aa position.

G) Boxplot of the individual aa-score of ZYX[223]_N, which is calculated using the new reference. The centerline in the boxplot depicts the median, the box limits depict the upper and lower quartiles, and the whiskers encompass 1.5 times the interquartile range (IQR). Statistical analysis was performed using Wilcox test for the comparison of two groups. "n" indicates the number of samples.


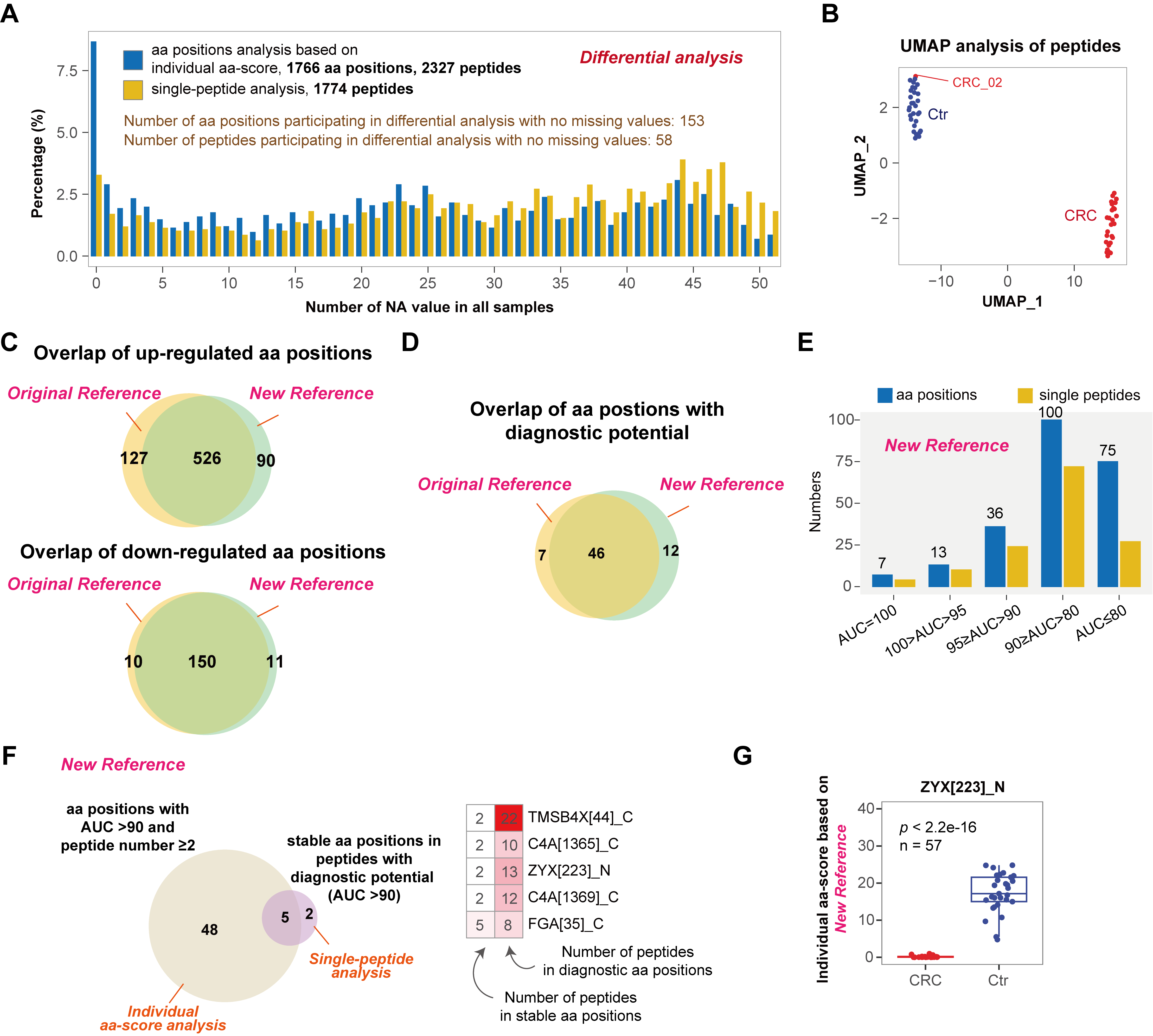

Supplement: Supplementary file 1 — Supporting Information [file ADVS-13-e10910-s001.docx]
